# New inorganic inhibitors derived from cefotaxime to enhance corrosion resistance of mild steel in 3% NaCl

**DOI:** 10.1038/s41598-024-51275-5

**Published:** 2024-01-10

**Authors:** Mustafa S. Abd El-Zahir, Mohamed H. A. Soliman, Hamdy A. ELKady, Sahar S. A. El-Sakka, Adel S. Orabi

**Affiliations:** 1 Department of Refining and Petrochemicals Engineering, Faculty of Petroleum and Mining Engineering, Suez University, P.O. Box: 43221, Suez, Egypt; 2 Department of Chemistry, Faculty of Science, Suez University, P.O. Box: 43221, Suez, Egypt; 3 Department of Chemistry, Faculty of Science, Suez Canal University, Ismailia, 41522 Egypt

**Keywords:** Organometallic chemistry, Chemical engineering

## Abstract

To overcome the threat of corrosion and its cost, a new Schiff base was prepared and utilized to synthesize inorganic inhibitors to enhance corrosion resistance and reduce current density. The Schiff base was obtained from the interaction of cefotaxime with acetylacetone, while ^1^H NMR and IR spectra were used to confirm the preparation. Moreover, Fe^III^, Co^II^, Ni^II^ and Cu^II^ metal salts were reacted with the Schiff base to give the corresponding complexes. Meanwhile, the non-ionic behavior of the observed complexes in solutions was proved from the conductance results. In addition, the octahedral geometry and the postulated structure of complexes were determined from CHNM%, IR spectroscopy, UV-visible spectra, and TGA analysis. Also, the energy of molecular orbitals (HOMO and LUMO) and other quantum mechanics parameters were calculated using the DFT method. The observed results indicated the reactivity of metal complexes and their ability to donate electrons more than the Schiff base. Furthermore, the corrosion rate of a steel sample under various concentrations of inhibitors was calculated by a potentiodynamic polarization test. The obtained data displayed that metal complexes declined the corrosion rate more than the Schiff base; therefore, the binding between the metal ion and the Schiff base improved the inhibition efficiency.

## Introduction

Corrosion is a chemical process, and it occurs when a material interacts destructively with its environment^1^. Thus, it is a major concern that affects the entire world due to the threat of financial losses caused by process interruptions, decreased productivity, and costly maintenance for repairing corroded buildings, mending damaged equipment, and replacing some of its parts^2^.

Corrosion can be successfully managed by understanding and knowing the mechanisms involved in the process. This covers material selection as well as environmental, temperature, and general conditions under which the material is employed. Therefore, a safer environment can be created by removing factors that promote corrosion, changing the temperature, removing moisture and oxygen, regulating the pH, or adding inhibitors and avoiding the connection between dissimilar metals^3,4^.

The use of inhibitors is one of the most effective corrosion prevention techniques, and several compounds have been tested for protecting metals^5,6^. Nitrogen compounds such as heterocyclic molecules and Schiff bases, sulfur organic compounds, which include sulphonamides that have numerous active groups, and oxygen and phosphorus compounds are examples of corrosion inhibitors^7–12^. These chemicals are applied in small quantities to resist corrosion, and they can protect the surface by reacting with the metal’s surface and forming an adhering coating or by reacting with the surrounding environment^13,14^.

Therefore, inhibitors can be attached to the surface of a metal by a variety of mechanisms, including complexation, adsorption (physical or chemical), and precipitation. This coating limits the corrosion process by blocking cathode reactions that involve hydrogen evolution or oxygen reduction to water, or by inhibiting metal dissociation into ions at the anode. Inhibitors can also reduce ion propagation over the metal surface and increase material resistance^15–18^. Additionally, the inhibitor constituents, structure, volume, the presence of heteroatoms, and quantum chemical parameters are factors that influence the efficiency of the inhibitor, while the inhibitor efficiency (IE%) may be calculated using experimental data acquired using methods such as potentiodynamic polarization^19^.

Recently, interaction between transition metals and inhibitors containing heteroatoms has been utilized to enhance corrosion inhibition. In this regard, E. Salehi and his co-workers reported a significant effect of the complexation of Zn^II^ with different organic inhibitors on the corrosion rate. Besides, other researchers investigated the effect of combining rare metals with organic inhibitors on the corrosion rate of mild steel^20–23^. Thus, this research aimed to prepare inorganic inhibitors based on transition metals to decline the corrosion rate of steel. Furthermore, spectroscopic and thermal studies were used to analyze the synthesized compounds. Meanwhile, a potentiodynamic polarization test was performed on a steel sample to display the corrosion inhibition effect.

## Experimental

### Material and methods

Methanol, acetylacetone, chloride salts of ferric, cobalt, and nickel, Cu(NO_3_)_2_∙6H_2_O, and DMSO were purchased with high purity, while cefotaxime was obtained from EIPICO Company. Adwa AD8000, spectroscopic instruments (atomic absorption, ALPHA II FTIR, Evolution™ 200 UV-vis, and Avance III NMR), and a Vario EL III elemental analyzer were utilized to characterize the compounds. Meanwhile, the Sherwood balance displayed the magnetic susceptibility of metal complexes. Furthermore, the TGA study was achieved under nitrogen by a Shimadzu analyzer at a rate of 20 °C min^−1^.

### Syntheses

#### Ligand preparation

**Figure 1:**
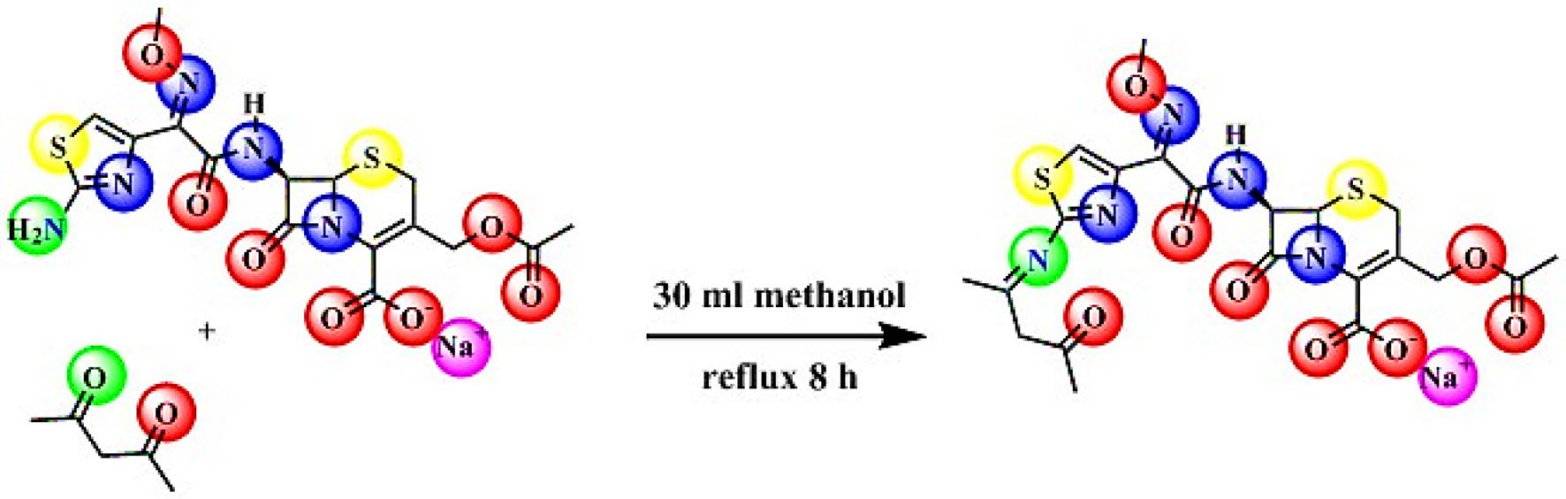
Schiff base synthesis from cefotaxime sodium salt and acetylacetone.

The Schiff base was prepared by mixing 1 mmol of acetylacetone dissolved in 10 ml of methanol with a 30 ml solution of cefotaxime (477 mg) in methanol for 8 h under reflux (Fig. 1).

#### Synthesis of metal complexes

**Figure 2:**
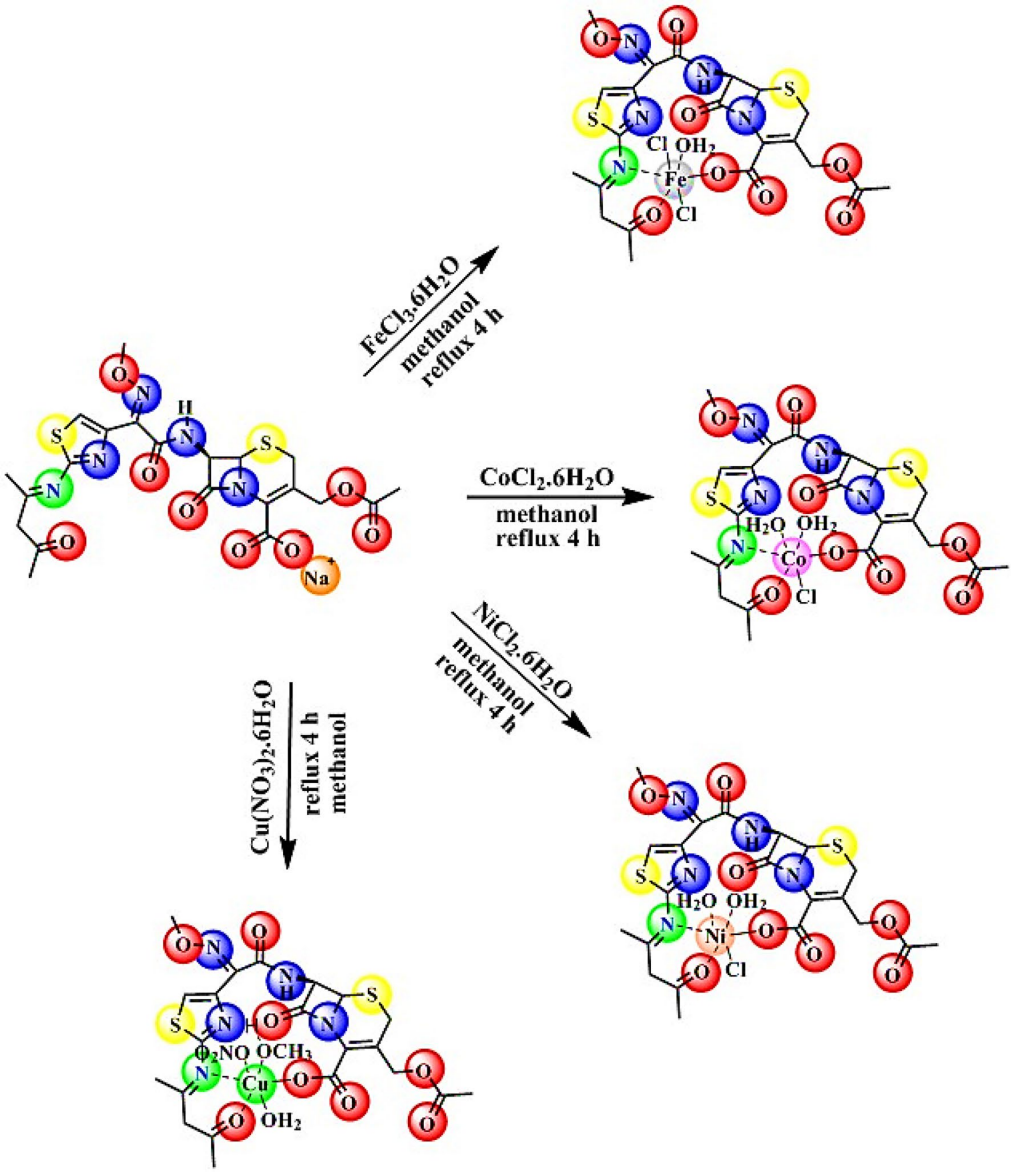
Preparation of metal complexes from Schiff base.

10 ml of metal ion solution (Fe^III^, Co^II^, Ni^II^, and Cu^II^) in methanol was reacted with the Schiff base under reflux for 4 h (Fig. 2), and it was noticed that the complex was precipitated after the addition of metal ion to the Schiff base solution. After filtration, the precipitate was washed several times with methanol and water and then kept in a desiccator.

### Quantum mechanics parameters

The geometry optimization and the energy minimization of the compounds were performed using the Gaussian 09W software package by the DFT method with the B3LYP hybrid functional and the 6-31G (d,p) basis set. To estimate the molecule’s stability, frontier orbitals (HOMO and LUMO) analysis was performed, and energy gap (ΔE), ionization energy (I), electronic affinity (A) were calculated using the energy values of highest occupied molecular orbital (HOMO) and lowest unoccupied molecular orbital (LUMO). Furthermore, softness (σ), chemical potential (Pi), electronegativity (χ), absolute hardness (η), Global softness (S), Global electrophilicity (ω) and additional electronegativity (ΔN_max_) were calculated.

$$\Delta {\text{E}} = {\text{ E}}_{{{\text{LUMO}}}} - {\text{E}}_{{{\text{HOMO}}}} \;\;\; {\text{I}} = - {\text{E}}_{{{\text{HOMO}}}} \;\;\; {\text{A}} = - {\text{E}}_{{{\text{LUMO}}}}$$

$$\eta = \frac{{{\text{E}}_{{{\text{LUMO}}}} { } - {\text{ E}}_{{{\text{HOMO}}}} }}{2}\;\;\;\chi = - \frac{{{\text{ E}}_{{{\text{HOMO}}}} + E_{{{\text{LUMO}}}} }}{2}$$

$$\sigma = \frac{1}{{\upeta }}\;\;{\text{S}} = \frac{1}{{2{{ \eta }}}}\;\;{\text{Pi }} = - \chi \;\;\omega \, = \frac{{{\text{P}}_{i}^{2} }}{{2{{ \eta }}}}\;\;\Delta {\text{N}}_{{{\text{max}}}} = - \frac{{{\text{P}}_{i} }}{{{{ \eta }}}}$$

### Potentiodynamic polarization method

**Figure 3:**
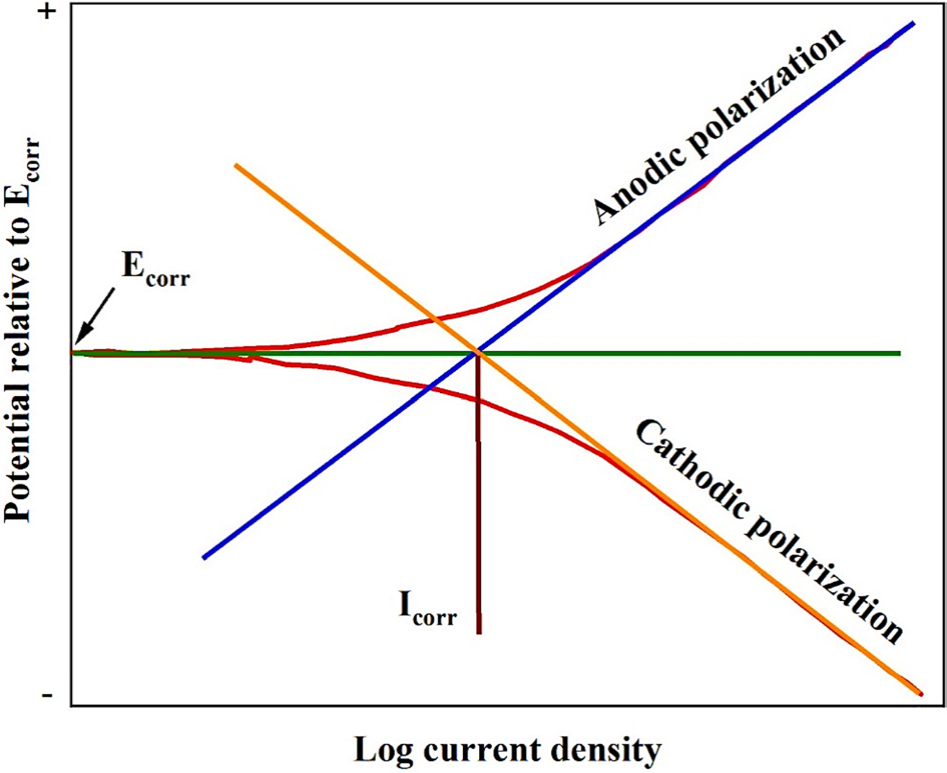
Polarization curve.

The polarization curve was obtained from the relation between the electrode potential and the logarithm of the resulting current by changing the electrode potential, and the resulting current was recorded. The polarization plot (Fig. 3) consists of three parts: the cathodic section is the lower part of the curve in which the sample electrode receives a high flow of electrons for the reduction reaction; thus, the sample potential moves toward negative values of corrosion potential. Meanwhile, the upper section displays the anodic polarization, where the sample is oxidized by passing an electric current in a direction opposite to the electron flow at the cathodic section. Therefore, the electrode potential has a positive value, while both sections are connected at a point called corrosion potential (**E**_**corr**_), which represents the sample potential during the loss of electrons to the environment and in the absence of an external current.

Potentiodynamic studies were carried out using a VersaSTAT-3 instrument to evaluate the impact of the Schiff bases and its metal complexes on the corrosion rate of steel. The VersaStudio software package was utilized to control the hardware by entering the input parameters and recording the polarization curves. Moreover, the experiments were carried out in a cell consisting of three electrodes: a working electrode (steel), a counter electrode (platinum), and a saturated calomel electrode, while these electrodes were immersed in a 3% NaCl solution. The polarization curves were determined by automatically changing the electrode potential from − 1.6 to + 1.6 V versus the open circuit voltage at a scan rate of 2 mV S^−1^, and the resulting current was recorded. The steel’s corrosion rate in 3% NaCl solution (blank solution) was recorded before and after the addition of Schiff base and its metal complexes. Furthermore, the experiment was performed with various concentrations of Schiff base and its metal complexes (10–40 ppm) to measure the impact of increasing concentrations on corrosion current, and each measurement took around half an hour to display the potentiodynamic scan.

The corrosion rates for the steel before and after the addition of inhibitors were calculated using the next equation:

$${\mathbf{Corrosion}}\; {\mathbf{rate}} \left( {{\mathbf{CR}}} \right) = \frac{{{\mathbf{0}}{\mathbf{.13}} \times {\mathbf{I}}_{{{\text{corr}}}} \user2{ } \times \user2{ }{\mathbf{At}}.{\mathbf{Wt}}}}{{\user2{\rho } \times \user2{ }{\mathbf{n}}}}$$

where, **I**_**corr**_, **At**.**Wt**, ***ρ*** and **n** are the corrosion current (μA cm^−2^), atomic weight of steel (55.845), density of steel (7.86 g cm^−3^) and valence of steel (n = 2), respectively. Meanwhile, the corrosion current (**I**_**corr**_) is the observed current at the intersection point of the extrapolated cathodic and anodic polarization lines with corrosion potential line.

Furthermore, the following equation was applied to estimate the inhibition efficiency (**IE**).

$${\mathbf{Inhibition}}\; {\mathbf{efficiency}} \left( {{\mathbf{IE}}} \right) = \frac{{{\mathbf{CR}}_{{{\mathbf{blank}}}} \times \user2{ }{\mathbf{CR}}_{{{\mathbf{inhibitor}}}} }}{{{\mathbf{CR}}_{{{\mathbf{blank}}}} }}\user2{ } \times \user2{ }100$$

## Results and discussion

It is clear that the metal–ligand ratio is 1:1 due to the M% values of all complexes, and the CHNM% data are shown in Table 1. Meanwhile, the solubility test for the metal complexes reveals that they only dissolve in DMSO. In addition, the conductivity values (Table 1) indicate the non-ionic structure of the inorganic compounds.

**Table 1:** Analytical data and conductivity of ligand and its complexes.

Formula	M.wt	Colour	Melting point (^o^C)	Elemental analysis				Conductivity ohm^−1 ^cm^−1 ^mol^−1^
				C%	H%	N%	M%	
				Found (Calc.)	Found (Calc.)	Found (Calc.)	Found (Calc.)	
Schiff base ligand L^−^Na^+^. (H_2_O)(CH_3_OH) (C_22_H_28_N_5_O_10_S_2_Na)	609.60	Orange	> 250	43.06 (43.35)	4.54 (4.63)	11.73 (11.49)	–	–
Iron III complex [Fe^III^L^−^(H_2_O)(Cl^−^)_2_](CH_3_OH)(H_2_O)	731.37	Deep brown	> 250	36.31 (36.13)	4.00 (4.13)	9.72 (9.58)	7.07 (7.64)	9.12
Cobalt II complex [Co^II^L^−^(H_2_O)_2_(Cl^−^)](H_2_O)	684.98	Brick brown	> 250	36.57 (36.82)	4.52 (4.12)	10.37 (10.22)	7.97 (8.60)	7.33
Nickel II complex [Ni^II^L^−^(H_2_O)_2_(Cl^−^)](CH_3_OH)	698.77	Faint green	> 250	37.59 (37.82)	4.57 (4.33)	10.36 (10.02)	7.75 (8.40)	7.02
Copper II complex [Cu^II^ L^−^(NO_3_^−^)(H_2_O)(CH_3_OH)](H_2_O)	730.18	green	> 250	35.88 (36.19)	3.90 (4.14)	11.73 (11.51)	8.28 (8.70)	7.69

### ^1^HNMR

**Figure 4:**
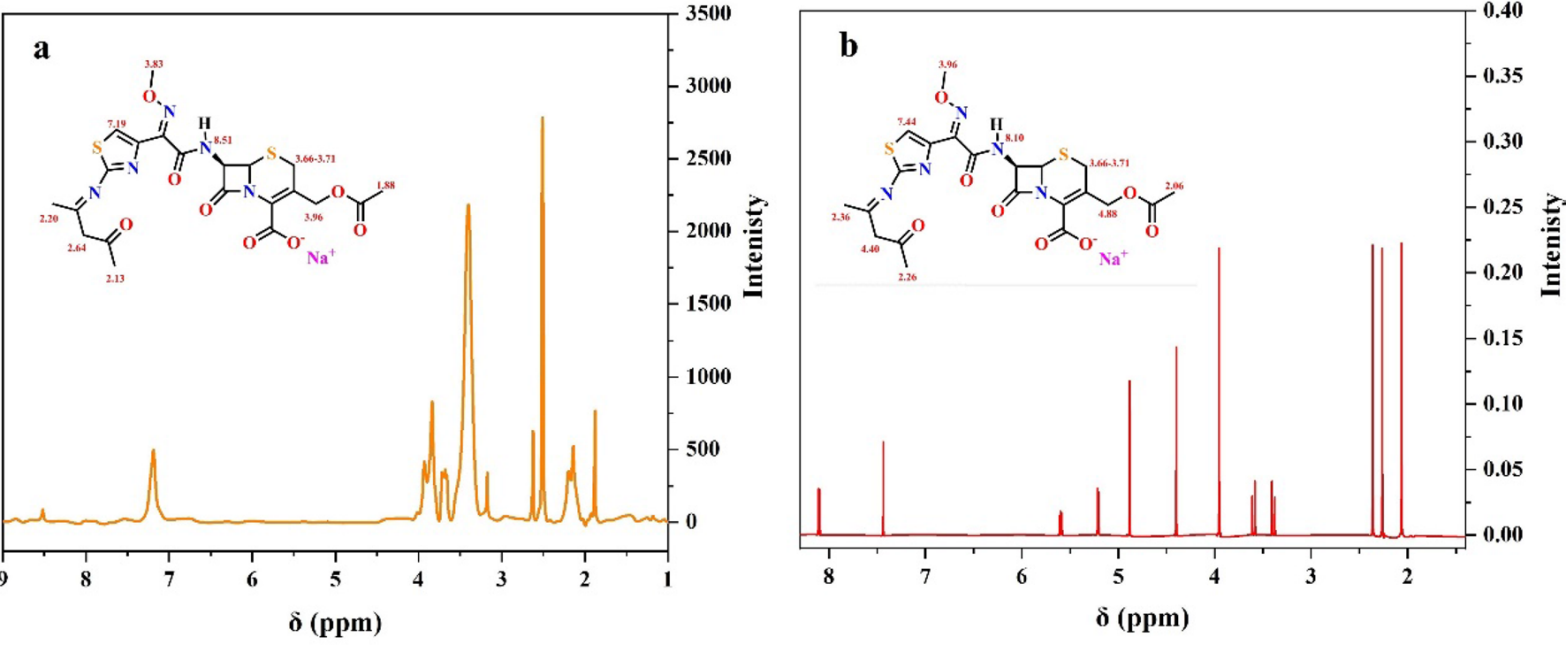
The experimental and calculated ^1^HNMR spectrum of the Schiff base.

Table 2 represents the data collected from the ^1^H NMR spectrum of the Schiff base 4-oxopentan-2-ylidene-cefotaxime using DMSO-*d*_*6*_ as a solvent in the range of 0–16 ppm. Meanwhile, the experimental and calculated spectra are shown in Fig. 4, and no signal is observed in the spectrum related to the presence of the cefotaxime amino group, supporting the formation of the Schiff base. Moreover, the ^1^H NMR spectrum of the Schiff base shows signals at *δ* 8.51 ppm and 7.19 ppm, which are assigned to the chemical shifts of the amide proton NH and the proton of the thiazole ring, respectively^24–26^. Also, signals assigned to OCH_2_ and OCH_3_ protons are observed in the regions of 3.96 and 3.83 ppm^25–27^, while signals characteristic for S-CH_2_ protons on the dihydrothiazine ring resonate in the 3.66–3.71 ppm region^28,29^. Furthermore, the signals related to –CH_2_– and methyl protons of the acetylacetone moiety and ester group are represented at δ 2.64, 2.20–2.13 and 1.88 ppm, respectively^24,30^.

**Table 2:** ^1^HNMR signals of the synthesized Schiff base.

Compound	δ (ppm)	
Schiff base ligand	8.51 (10H, s, NH), 7.19 (1H, s, –CH thiazol), 3.96 (2H, s, –O–CH_2_), 3.83 (3H, s, –OCH_3_), 3.66–3.71 (2H, AB, –S–CH_2_), 2.64 (2H, s, –CH_2_–), 2.20 (3H, s, N=C–CH_3_), 2.13 (3H, s, O=C–CH_3_), 1.88 (3H, s, CH_3_)	

	Experimental	Calculated

### FTIR

**Figure 5:**
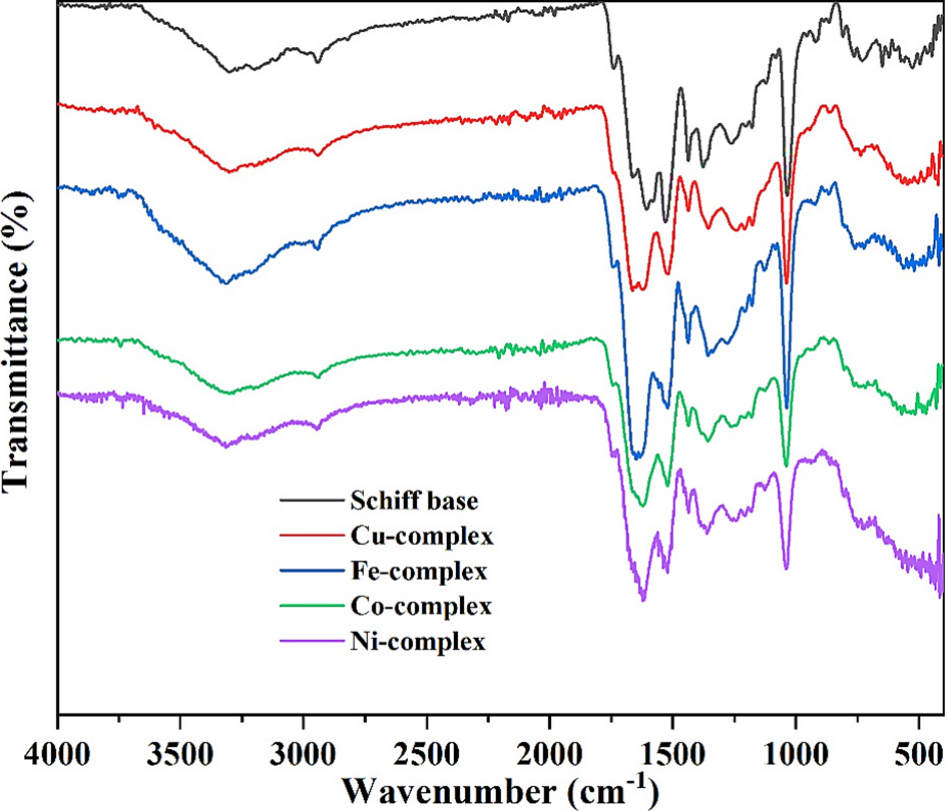
FTIR spectra of the formed Schiff base and its complexes.

The infrared spectra of the Schiff base and its complexes from Fe III, Co II, Ni II, and Cu II ion salts are displayed in Fig. 5, and the main vibration bands are shown in Table 3.

**Table 3:** FTIR spectra for the ligand and its metal complexes.

Compound	IR (cm^−1^)									
	ν(NH) amide	ν(C=O) β-lact	ν(C=O) ester	ν(C=N)	ν(C=O)*	ν (COO)_asy_	ν(COO)_sym_	Δν	ν(M–O)	ν(M–N)
Schiff base ligand L^−^Na^+^. (H_2_O)(CH_3_OH)	3305	1742	1662	1606	1530	1580	1377	–	–	–
Iron III complex [Fe^III^L^−^(H_2_O)(Cl^−^)_2_](CH_3_OH)(H_2_O)	3307	1743	1660	1624	1518	1557	1356	199	585	451
Cobalt II complex [Co^II^L^−^(H_2_O)_2_(Cl^−^)](H_2_O)	3300	1743	1662	1620	1520	1550	1357	193	587	455
Nickel II complex [Ni^II^L^−^(H_2_O)_2_(Cl^−^)](CH_3_OH)	3308	1739	1661	1622	1520	1558	1359	199	580	452
Copper II complex [Cu^II^ L^−^(NO_3_^−^)(H_2_O)(CH_3_OH)](H_2_O)	3301	1742	1662	1620	1520	1570	1355	215	588	454

*Acetylacetone.

The spectra of complexes show broad bands in the range of 3060–3700 cm^−1^ assigned to ν (N–H) and the vibration of water molecules, while the ν (N–H) is observed at 3307, 3300, 3308 and 3301 cm^–1^ for Fe(III), Co(II), Ni(II), and Cu(II) complexes, respectively, and these values are similar to the band position of the Schiff base, which appears at 3305 cm^−1^. Moreover, the vibration stretching band of the lactam carbonyl group appears at 1742 cm^−1^ for the Schiff base and copper complex, at 1739 cm^−1^ for nickel complex, and at 1743 cm^−1^ for both cobalt and iron complexes^24,31^. Meanwhile, ν (C=O) amide shows an absorption at 1648 cm^−1^ for the Schiff base^25,32^. Thus, there is almost no change in the ν (C=O) lactam and ν (C=O) amide bands position of metal complexes compared to the Schiff base, confirming that these groups are not involved in complex formation.

**Figure 6:**
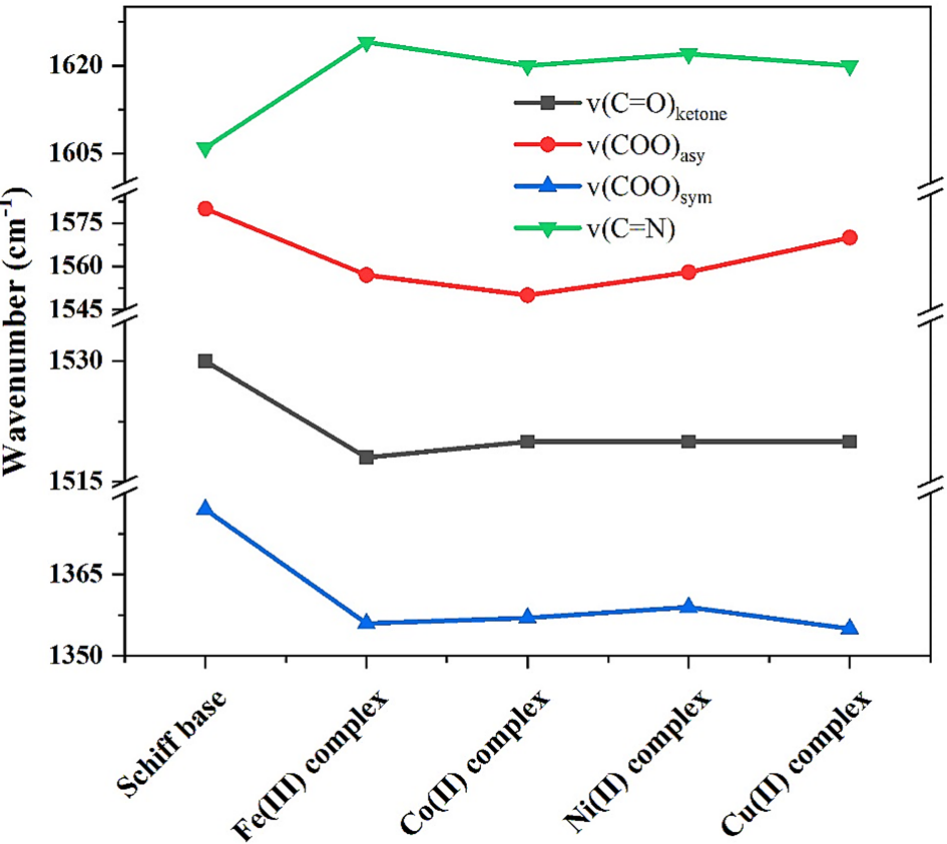
IR frequency change of imine, carboxylate and carbonyl group in acetylacetone for Ligand 3 and its complexes.

In addition, the groups involved in coordination are detected by observing the frequency change of their bands in the spectra of the complexes when compared to the ligand vibration values (Fig. 6). In Table 4, the two vibration bands of the carboxyl group for asymmetric and symmetric stretching are observed at lower frequencies in the complexes compared to the Schiff base^32^. Besides, the vibration bands of the carbonyl group of acetylacetone at 1530 cm^−1^ and the imine group ν (C=N) in the Schiff base are shifted in all metal complexes^33–35^. Meanwhile, the monodentate nature of the carboxyl group in coordination with the metal ion is expected because of the large separation distance between the two carboxyl bands (ν (COO)_asym_ and ν (COO)_sym_)^36^.

**Table 4:** The electronic spectra of the Schiff base and its metal complexes.

Compound	Peak		assignment	μ_eff_	Proposed structure
	nm	cm^−1^			
Schiff base ligand	230 299	43,478 33,445	π → π* n → π*	–	–
Iron III complex [Fe^III^L^−^(H_2_O)(Cl^−^)_2_](CH_3_OH)(H_2_O)	232 298 962	43,103 33,557 10,395	π → π* n → π* ^6^A_1g_ (S) → ^4^T_1g_ (G)	5.43	O_h_
Cobalt II complex [Co^II^L^−^(H_2_O)_2_(Cl^−^)](H_2_O)	232 294 758 918	43,103 34,014 13,193 10,893	π → π* n → π* ^4^T_1g_ → ^4^A_2g_ ^4^T_1g_ → ^4^T_2g_	4.17	O_h_
Nickel II complex [Ni^II^L^−^(H_2_O)_2_(Cl^−^)](CH_3_OH)	232 297 728 926	43,103 33,670 13,736 10,799	π → π* n → π* ^3^A_2g_ → ^3^T_1g_ (F) ^3^A_2g_ → ^3^T_2g_	3.32	O_h_
Copper II complex [Cu^II^ L^−^(NO_3_^−^)(H_2_O)(CH_3_OH)](H_2_O)	245 308 772 939	40,816 32,468 12,953 10,650	π → π* n → π* ^2^B_1g_ → ^2^B_2g_ + ^2^B_1g_ → ^2^E_g_ ^2^B_1g_ → ^2^A_1g_	2.25	O_h_

Furthermore, the weak stretching bands around 451–455 and 580–588 cm^−1^ are assigned as M–N and M–O bonds, respectively.

### UV–Vis spectra

**Figure 7:**
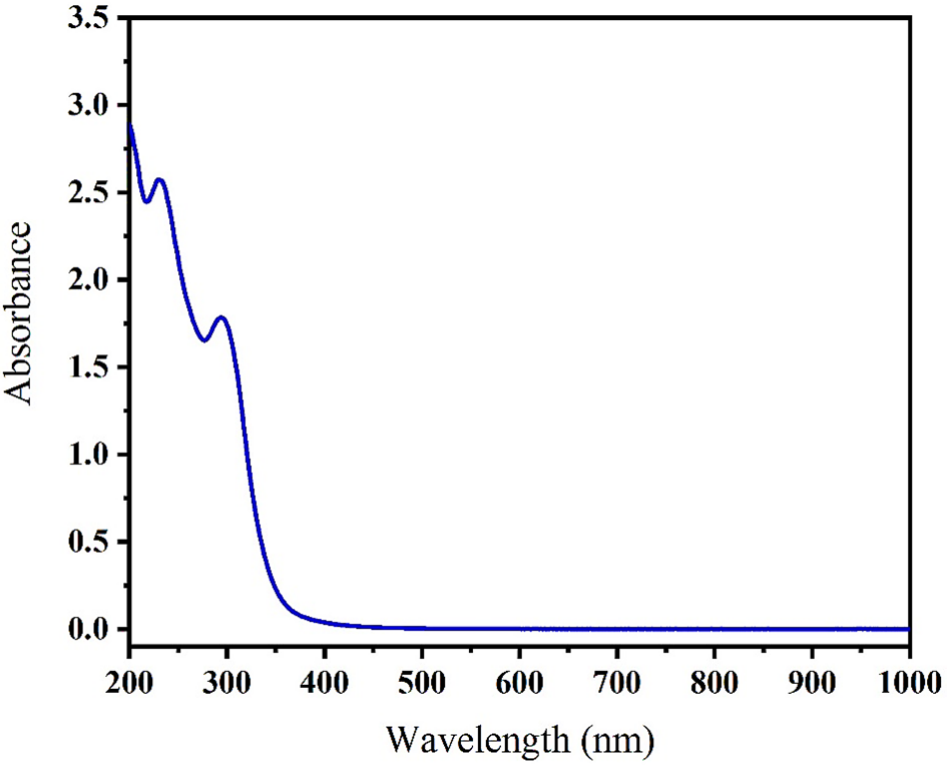
Electronic transition spectrum of the Schiff base.

**Figure 8:**
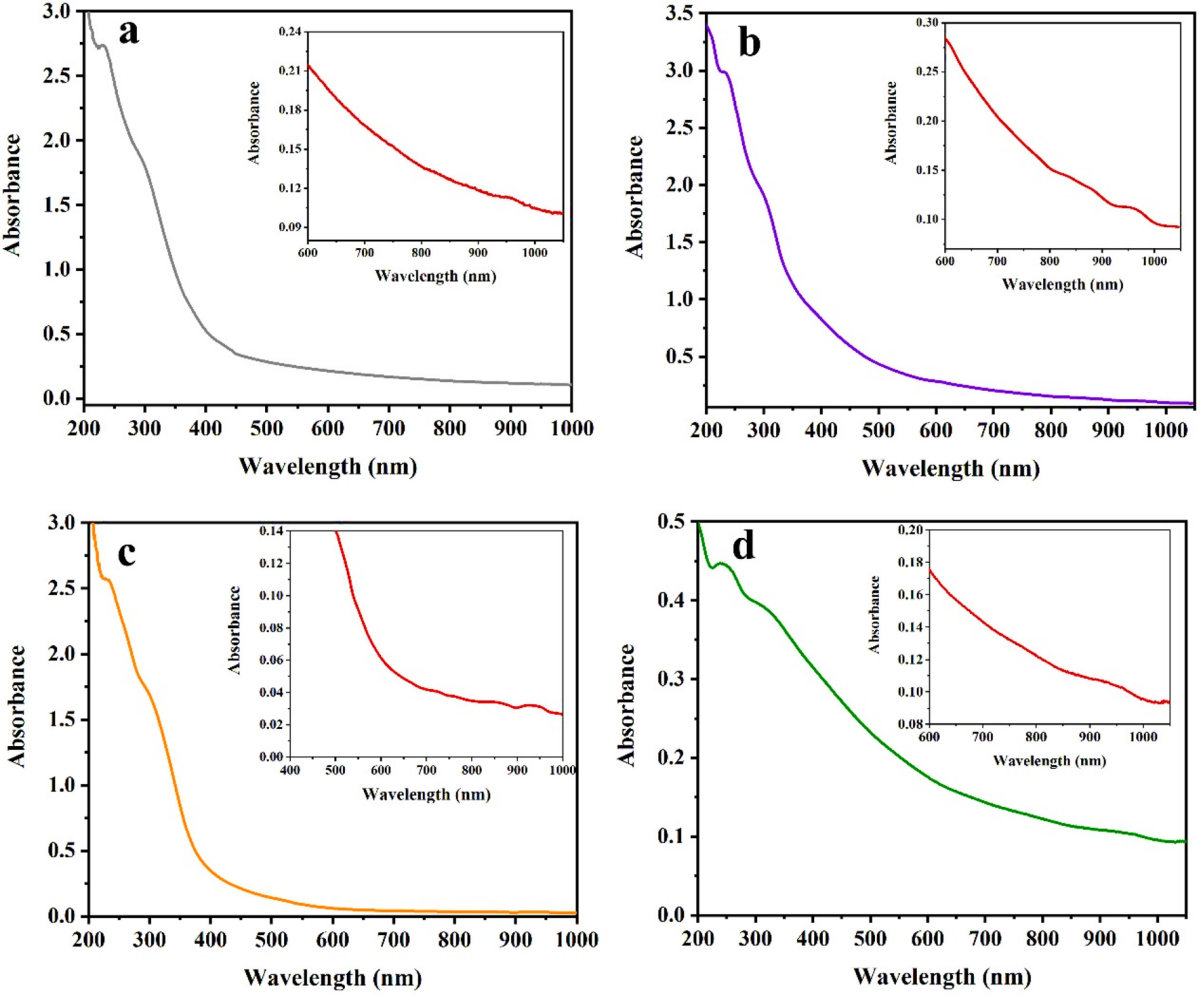
Electronic transition spectra of the complexes: (**a**) Fe-complex, (**b**) Co-complex, (**c**) Ni-complex and (**d**) Cu-complex.

The UV–visible spectra of the Schiff base and its complexes are displayed in Figs. 7, 8, and the observed data are represented in Table 4. The bands of Schiff base with respect to π–π* and n–π* are detected at 43,478 cm^−1^ and 33,445 cm^−1^, respectively (Fig. 7), while the magnetic susceptibility, which measures the magnetic behavior of the compounds (Table 4), exhibited that the complexes are distinguished as having a high-spin octahedral geometry with a sp3d2 configuration (outer complex).

The UV–visible spectrum of the **iron complex** (Fig. 8a) exhibits a transition band at 10,395 cm^−1^, which refers to ^6^A_1g_ (S) → ^4^T_1g_ (G), while the value of the magnetic moment is 5.43 BM, which is consistent with the geometry of octahedral compounds^37,38^. Meanwhile, Fig. 8b displays the electronic transitions of the **cobalt complex**, showing the wavelength of the bands at 758 nm (13,193 cm^−1^) and 918 nm (10,893 cm^−1^), which are attributable to the d-d transitions ^4^T_1g_ → ^4^A_2g_ and ^4^T_1g_ → ^4^T_2g_, respectively. Moreover, 4.17 BM is the observed value of the magnetic moment, which proves the octahedral structure of the complex^39^.

In the case of the** nickel complex**, the paramagnetic behavior of an octahedral spin configuration is confirmed by the magnetic moment, which is found to be 3.32 BM. Meanwhile, the electronic transitions of the spectrum (Fig. 8c) show bands for ^3^A_2g_ → ^3^T_1g_ (F) and ^3^A_2g_ → ^3^T_2g_ transitions at 728 nm (13,736 cm^−1^) and 926 nm (10,799 cm^−1^), respectively^40,41^. Furthermore, the magnetic moment and d-d transition bands from Fig. 8d reveal the octahedral structure of the **Cu complex**^42–44^.

### Thermal analysis

**Figure 9:**
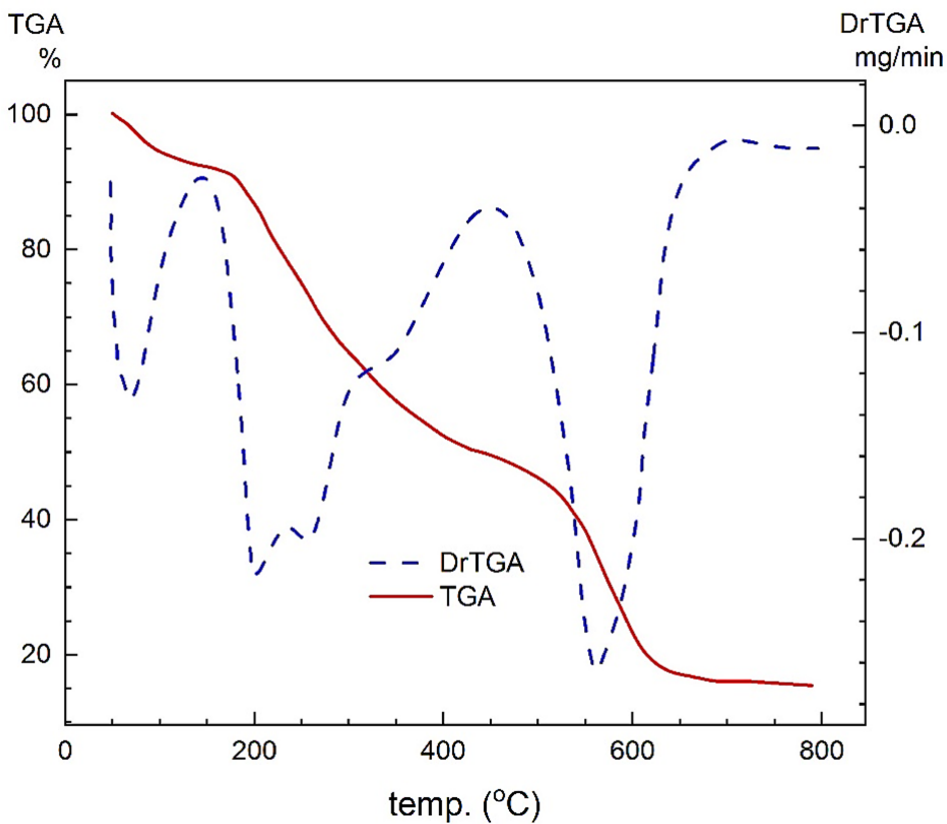
Thermal analysis graph of the Schiff base.

**Figure 10:**
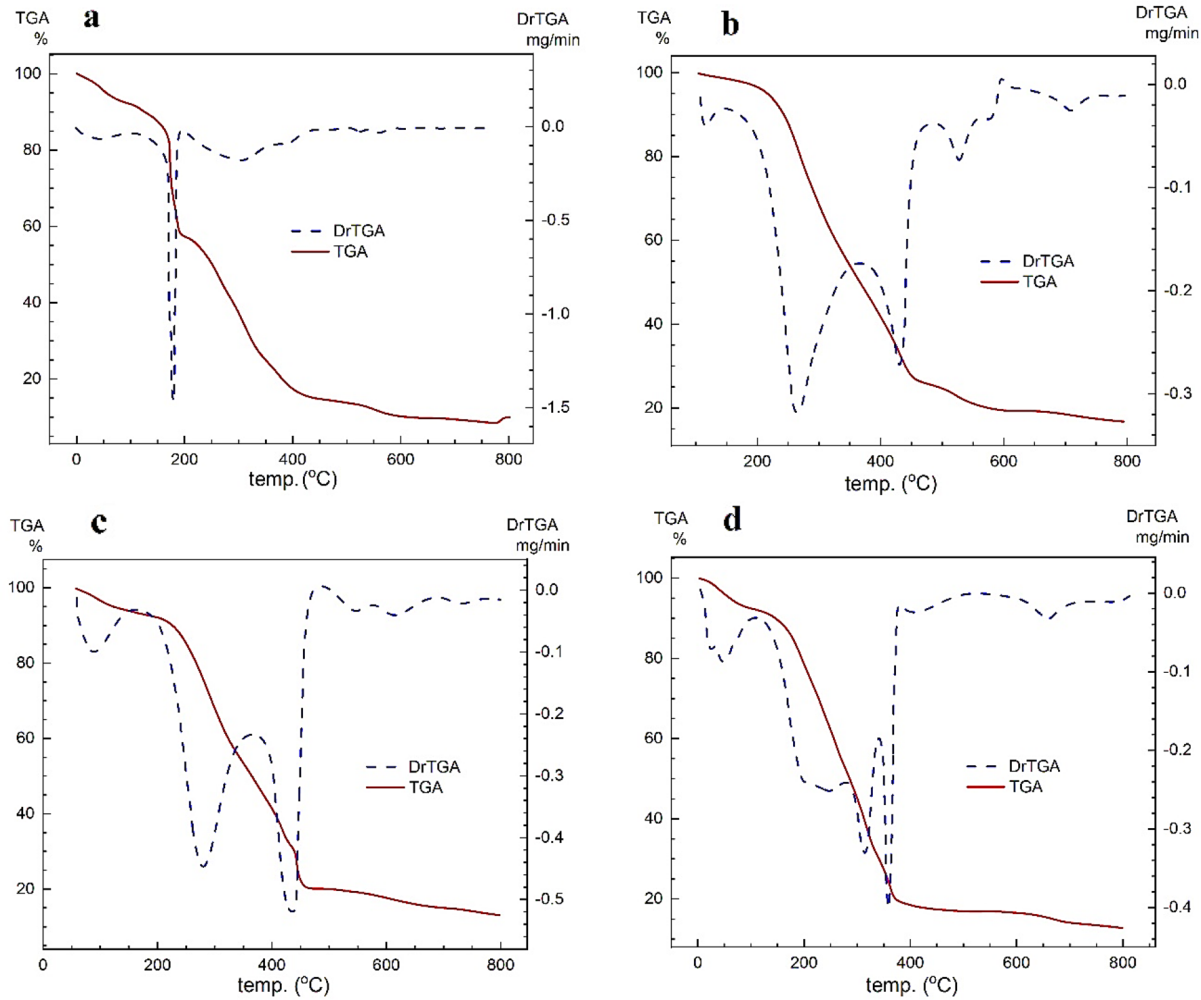
TGA graphs of metal complexes: (**a**) Fe-complex, (**b**) Co-complex, (**c**) Ni-complex and (**d**) Cu-complex.

**Figure 11:**
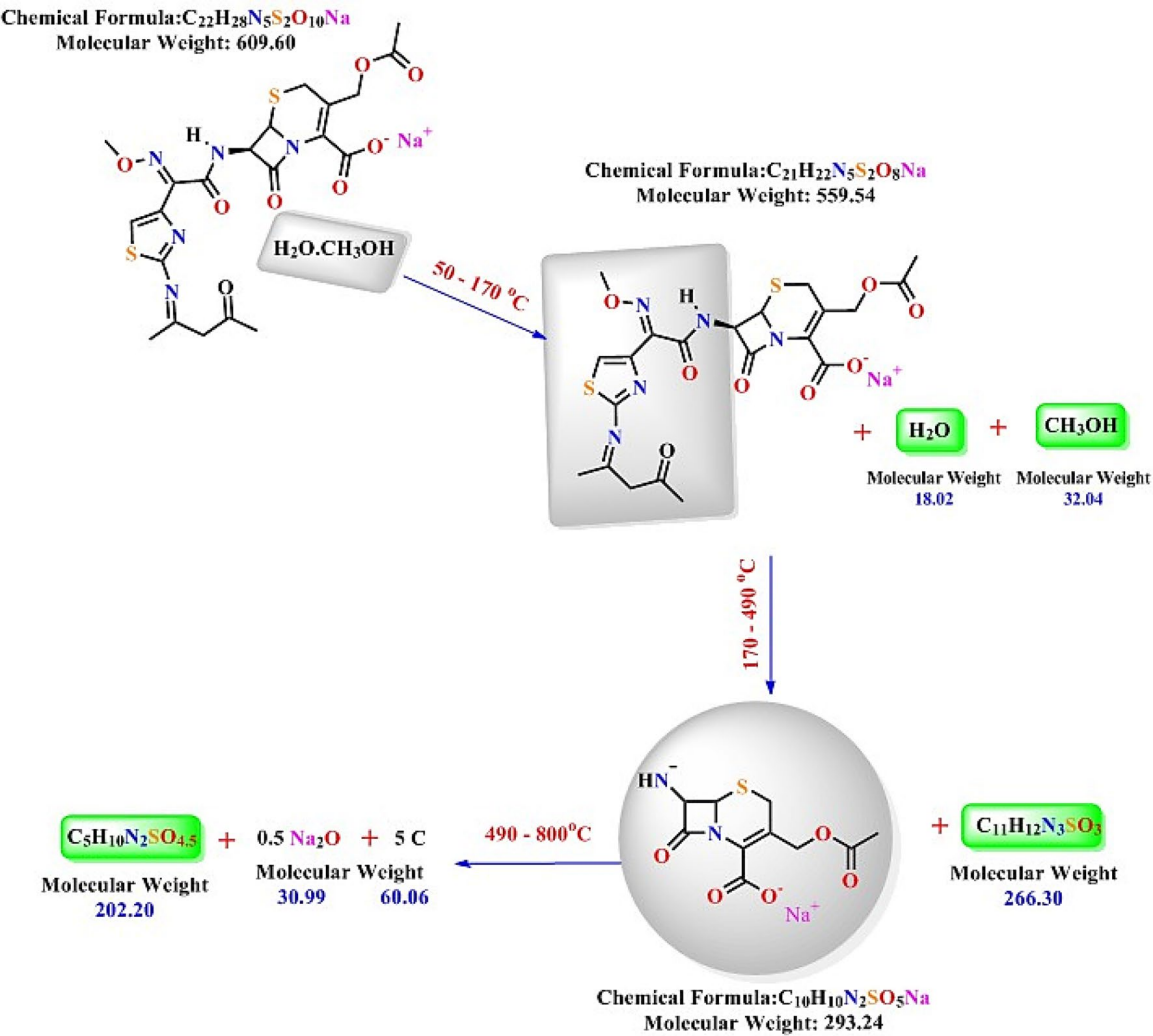
Postulated thermal decomposition of the Schiff base.

Figures 9 and 10 exhibit the thermal analyses of the Schiff base and its metal complexes, and the data are shown in Table 5. Figure 9 shows that the Schiff base is decomposed in three steps. In the first stage, the liberation of methanol and water molecules is observed, with a loss of 8.24% of the Schiff base weight (Calcd. = 8.21%) between 50 and 170 °C, while the ligand begins to decompose at 170 °C up to 800 °C during the second and third steps (Fig. 11). The second stage, from 170 °C to 490 °C, involves the decomposition of the side chain of the β-lactam ring from the amide bond, which includes the 2-(methoxyimino)acetyl and thiazol rings, and shows a weight loss of 43.76% (Calcd. = 43.68%). Meanwhile, complete decomposition of the ligand appears at the third step with a weight loss of 33.00% (Calcd. = 33.17%) within the temperature range of 490–800 °C. Furthermore, it is observed that the remainder of the Schiff base is Na_2_O and carbon with a mass loss of 15.00%, which is in agreement with the calculation (Calcd. = 14.93%).

**Table 5:** Thermal analysis of the Schiff base and its metal complexes.

Compound	Steps	Temp. Range ^o^C	Mass loss %		Decomposition process	Residue
			Found	Calcd		Found (Calcd. %)
Schiff base ligand L^−^Na^+^. (H_2_O)(CH_3_OH)	I	50–170	8.24	8.21	CH_3_OH + H_2_O	0.5 Na_2_O + 5C 15 (14.93)
	II	170–490	43.76	43.68	C_6_H_4_N_2_O_2_S + C_5_H_8_NO	
	III	490–800	33.00	33.17	C_5_H_10_N_2_O_4.5_S	
Iron III complex [Fe^III^L^−^(H_2_O)(Cl^−^)_2_](CH_3_OH)∙(H_2_O)	I	10–80	6.88	6.84	H_2_O + CH_3_OH	FeO 9.59 (9.82)
	II	80–195	35.75	35.56	H_2_O + C_8_H_13_NO_3_ + Cl_2_	
	III	195–800	47.78	47.77	C_13_H_9_N_4_O_4_S_2_	
Cobalt II complex [Co^II^L^−^(H_2_O)_2_(Cl^−^)](H_2_O)	I	50–451	72.50	72.69	3H_2_O + C_17_H_19_N_4_O_6_S + HCl	CoS + 2C 16.80 (16.79)
	II	451–800	10.70	10.52	C_2_H_2_NO_2_	
Nickel II complex [Ni^II^L^−^(H_2_O)_2_(Cl^−^)](CH_3_OH)	I	50–145	4.64	4.59	CH_3_OH	NiO + 2C 13.93 (14.13)
	II	145–455	75	74.99	2H_2_O + C_18_H_19_N_4_O_6_S_2_ + HCl	
	III	455–800	6.43	6.30	CH_2_NO	
Copper II complex [Cu^II^ L^−^(NO_3_^−^)(H_2_O)(CH_3_OH)](H_2_O)	I	10–105	6.77	6.86	CH_3_OH + H_2_O	CuO + 2C 14.20 (14.18)
	II	105–800	79.03	78.96	H_2_O + C_19_H_22_N_5_O_7_S_2_ + NO_3_^**-**^

In Fig. 10a, the thermal behavior of **Fe(III) complex** exhibits the liberation of CH_3_OH and H_2_O with a weight loss of 6.88% in its first stage of decomposition (Calcd. = 6.84%), and the following steps display the complete decomposition of the compound across two temperature regions. Temperature between 80 and 195 ^o^C involves a loss of coordinated H_2_O, HCl and acetoxymethyl moiety with a weight loss of 35.75% (Calcd. = 35.56%). Furthermore, the Schiff base is decomposed completely in the range of 195–800 °C with a mass loss of 47.78% (Calcd. = 47.77%), while the residual percentage is 9.59, which is compatible with the purposed value (Calcd. = 9.82%) for FeO.

In the case of the Co complex, Fig. 10b shows the complete thermal decomposition of the compound in two stages, where the first stage involves the liberation of water molecules and partial decomposition of the Schiff base with a mass loss of 72.50% (Calcd. = 72.69%) in the range of 50–451 °C. Meanwhile, the weight loss of 10.70% (Calcd. = 10.52%) appears at 451–800 °C, indicating complete decomposition of the ligand. Moreover, the remainder of the decomposition (16.80%) is attributed to the presence of cobalt sulfide (Calcd. = 13.28%) and carbon (Calcd. = 3.51%).

Regarding the** Ni complex** in Fig. 10c, the removing of methanol in the first step results in a weight loss of 4.64% (Calcd. = 4.59%), while the complex is decomposed in the range of 145–800 °C, which involves the liberation of coordinate H_2_O, HCl and complete ligand decomposition with a weight loss of 81.43% (Calcd. = 81.29%). Meanwhile, the residual of the complex (13.93%) is recognized as NiO (Calcd. = 10.69%) and carbon (Calcd. = 3.44%). In addition, the first step in Fig. 10d displays the presence of solvent molecules in **Cu-complex**, while CuO is observed in the complex residue (10.89%).

### Frontier molecular orbital

The technique of quantum mechanics is applied to describe the relationship between the geometry of compounds and their electronic characteristics, and then the electronic characteristics of the prepared Schiff base are compared with those of its complexes. Moreover, the study of molecular orbitals (HOMO and LUMO), also called frontier orbitals, is an essential step to elucidate various chemical parameters of compounds^45^. The energy of HOMO orbital shows its ability to lose electrons; thus, the ionization potential is connected with it, while the electronic affinity links to the energy of the LUMO orbital, which shows the ability to accept electrons^46–48^. Meanwhile, the difference between the two molecular orbitals, the “energy gap”, measures the reactivity of the molecule; the larger the energy gap, the lower the reactivity and polarizability of the compound^49,50^.

Additionally, the softness and hardness expressions are related to size, charge, and polarizability. Hence, it is clear that the hard ones are small in size, have a large charge, and are less polarized, while the soft ones are large in size, have a low charge, and are polarizable. Moreover, there is a relationship between the energy gap and the softness or hardness of molecules. Highly reactive molecules have a low energy gap, and they are soft because of their ability to donate electrons^51,52^. Meanwhile, electrophilicity is affected by hardness and chemical potential, and it is utilized to observe the lack of electrons and their reactivity towards the gain of electrons. Furthermore, chemical potential describes the ability to donate or accept electrons; thus, a compound with a small chemical potential is an electrophile, while a nucleophile displays high chemical hardness with a small negative value of chemical potential^53^.

**Figure 12:**
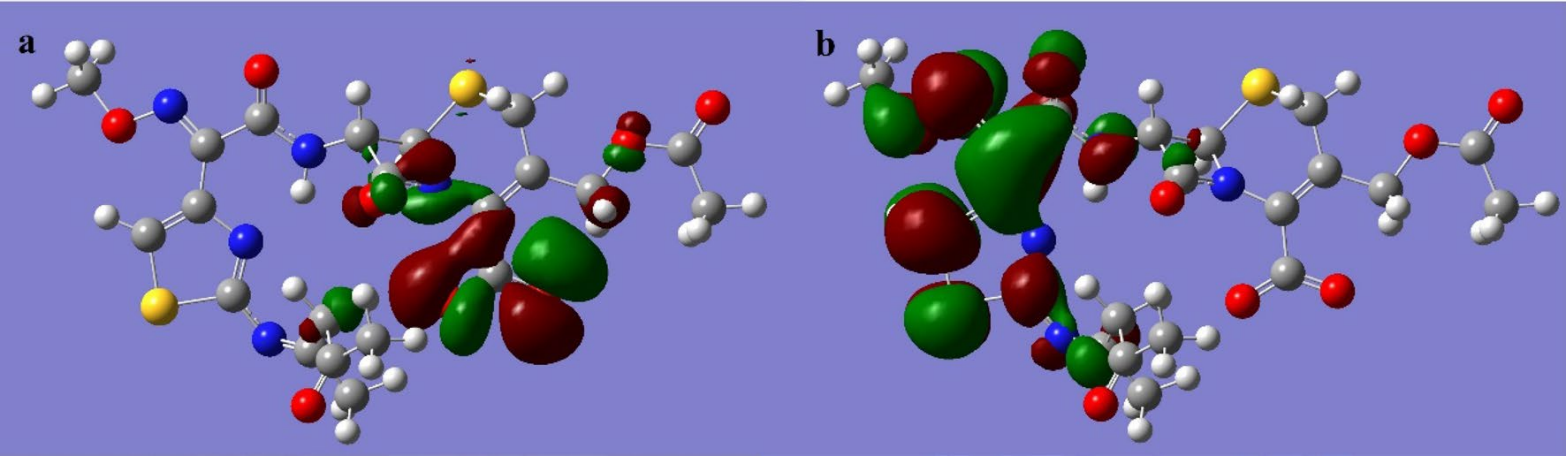
Schiff base HOMO and LUMO orbitals: (**a**) HOMO and (**b**) LUMO.

**Figure 13:**
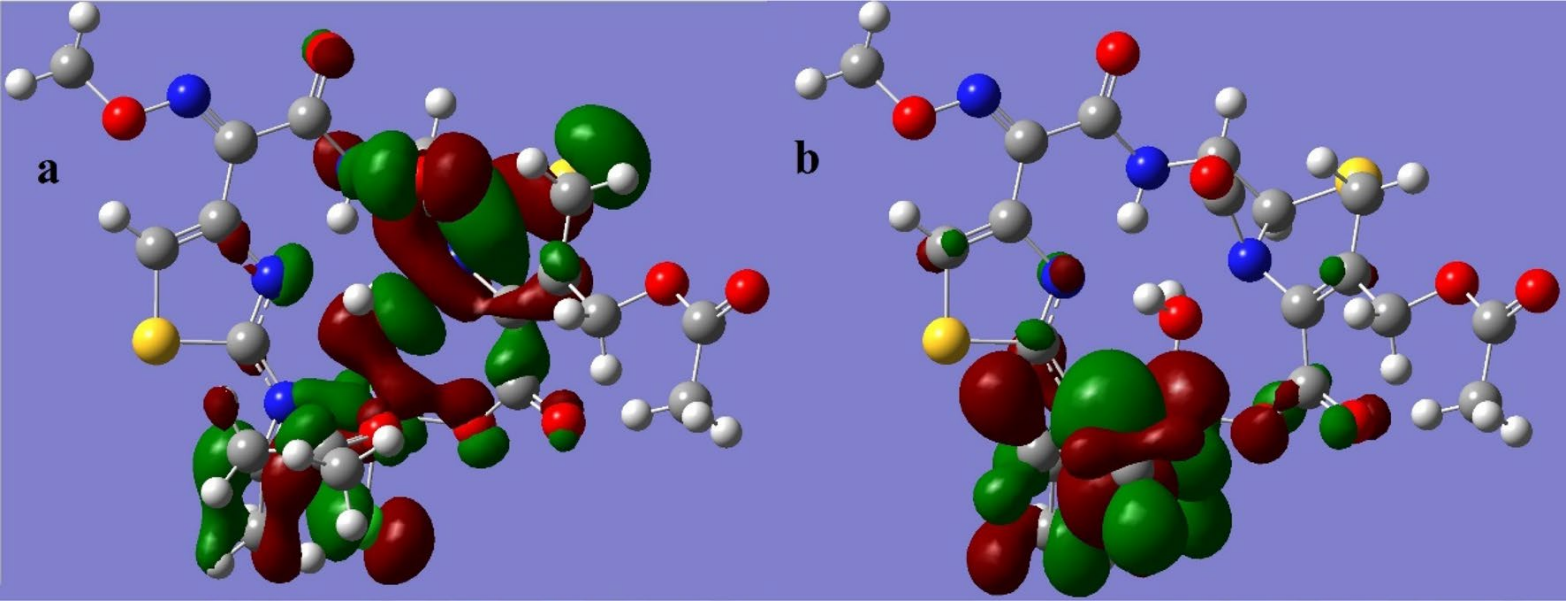
Fe complex HOMO and LUMO orbitals: (**a**) HOMO and (**b**) LUMO.

**Figure 14:**
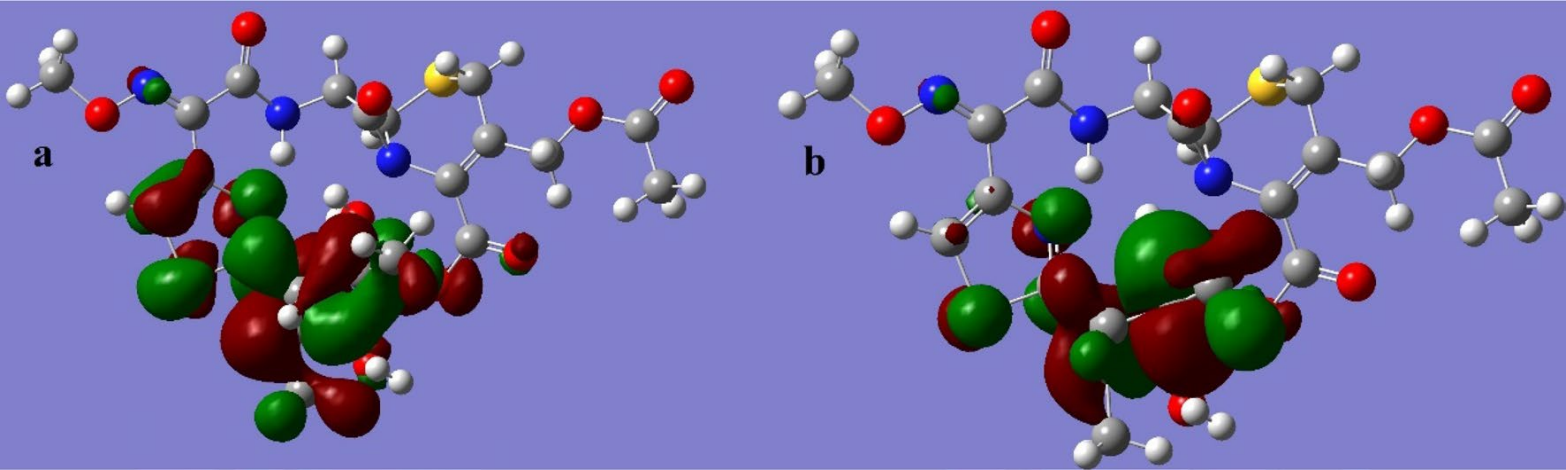
Co complex HOMO and LUMO orbitals: (**a**) HOMO and (**b**) LUMO.

**Figure 15:**
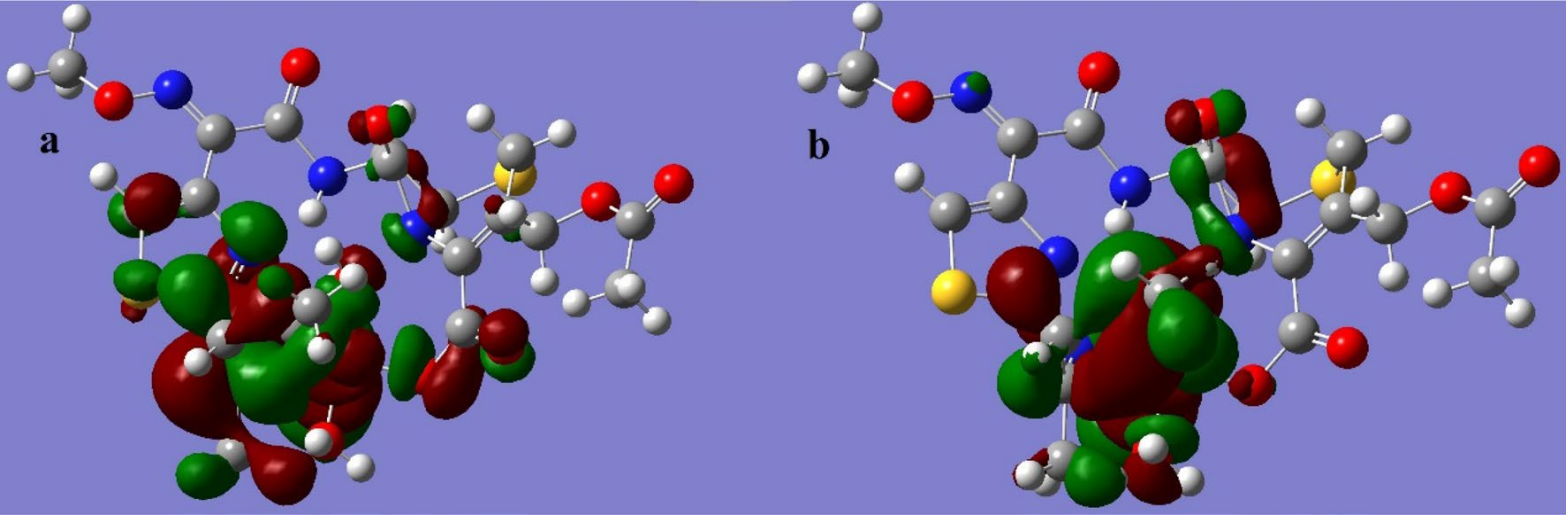
Ni complex HOMO and LUMO orbitals: (**a**) HOMO and (**b**) LUMO.

**Figure 16:**
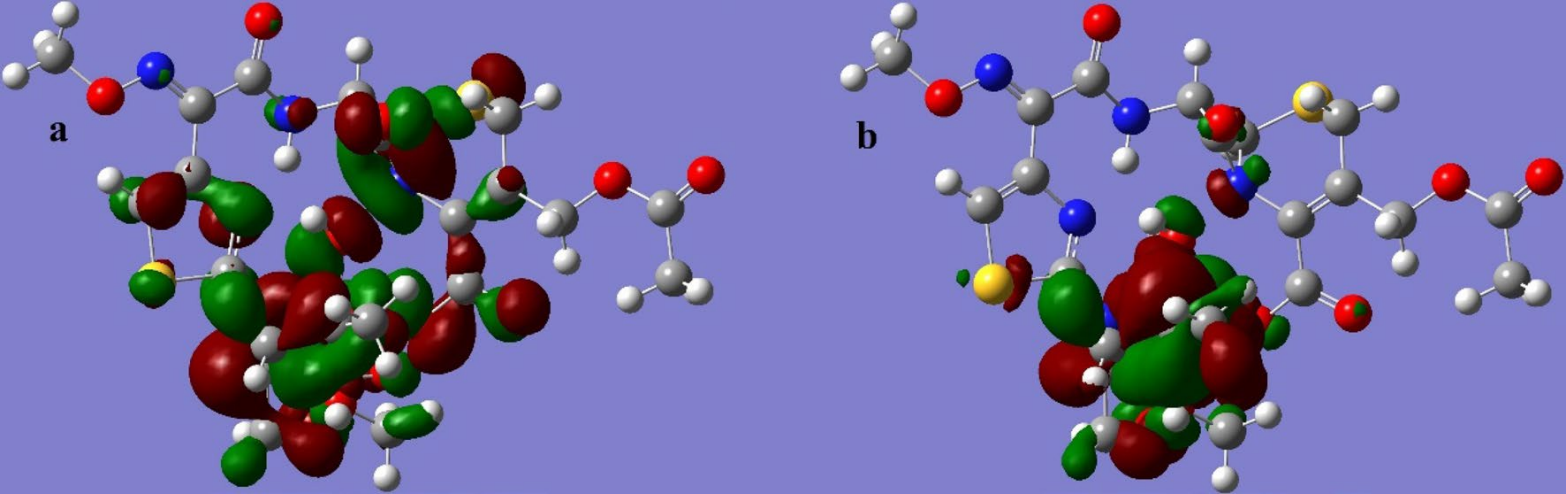
Cu complex HOMO and LUMO orbitals: (**a**) HOMO and (**b**) LUMO.

The LUMO and HOMO orbitals of the Schiff base and its complexes are represented on the basis of the DFT/B3LYP/6-31G (d,p) calculations using the Gaussian program in Figs. 12, 13, 14, 15 and 16. Moreover, energy gap (ΔE), ionization energy (I), electronic affinity (A), softness (σ), chemical potential (Pi), electronegativity (χ), absolute hardness (η), Global electrophilicity (ω), and additional electronegativity (ΔN_max_) are displayed in Table 6.

**Table 6:** Schiff base and its complexes quantum mechanics parameters.

Compound	Schiff base	Iron III complex	Cobalt II complex	Nickel II complex	Copper II complex
Formula	C_21_H_22_N_5_O_8_S_2_	C_21_H_24_Cl_2_FeN_5_O_9_S_2_	C_21_H_26_ClCoN_5_O_10_S_2_	C_21_H_26_ClNiN_5_O_10_S_2_	C_22_H_28_CuN_6_O_13_S_2_
Atoms	58	64	66	66	72
SCF energy	− 2485.34 au	− 4745.20 au	− 4480.63 au	− 4605.98 au	− 4597.41 au
Dipole	8.22 Debye	15.62 Debye	14.20 Debye	15.50 Debye	15.88 Debye
E_HOMO_	− 2.81 eV	− 0.19	− 0.19	− 0.17	− 0.19
E_LUMO_	0.48 eV	− 0.13	− 0.12	− 0.12	− 0.14
ΔE	3.28 eV	0.06	0.07	0.05	0.05
I (I.E)	2.81 eV	0.19	0.19	0.17	0.19
Electron affinity (A)	− 0.48 eV	0.13	0.12	0.12	0.14
Absolute electronegativity (χ)	0.93	0.23	0.21	0.20	0.23
Absolute hardness (η)	1.64	0.03	0.03	0.03	0.03
Absolute softness (σ)	0.61	0.02	0.02	0.01	0.01
Global softness (S)	0.30	15.80	14.48	18.14	18.25
Global electrophilicity (ω)	0.26	0.83	0.65	0.70	1.01
Chemical potential (Pi)	− 0.93	− 0.23	− 0.21	− 0.20	− 0.23
Additional electronegativity (ΔN_max_)	0.57	7.23	6.13	7.14	8.61

It is clear from Table 6 that the ionization potential is shown for all compounds, and it is 2.81 eV for the Schiff base, while for metal complexes, it ranges from 0.17 to 0.19 eV. Therefore, the Schiff base has the lowest ability to lose electrons. Meanwhile, the metal complexes show the highest electronic affinity value when compared to the Schiff base. In addition, the HOMO–LUMO energy gaps of the compounds are displayed in Table 6, and the lowest energy gap is shown in the Ni and Cu complex. Hence, these compounds are more chemically reactive and able to transfer electrons between orbitals more easily. Furthermore, the softness of the metal complexes and the hardness of the Schiff base are determined by the values of hardness (η), softness (S), and chemical potential (Pi).

### Potentiodynamic technique

**Figure 17:**
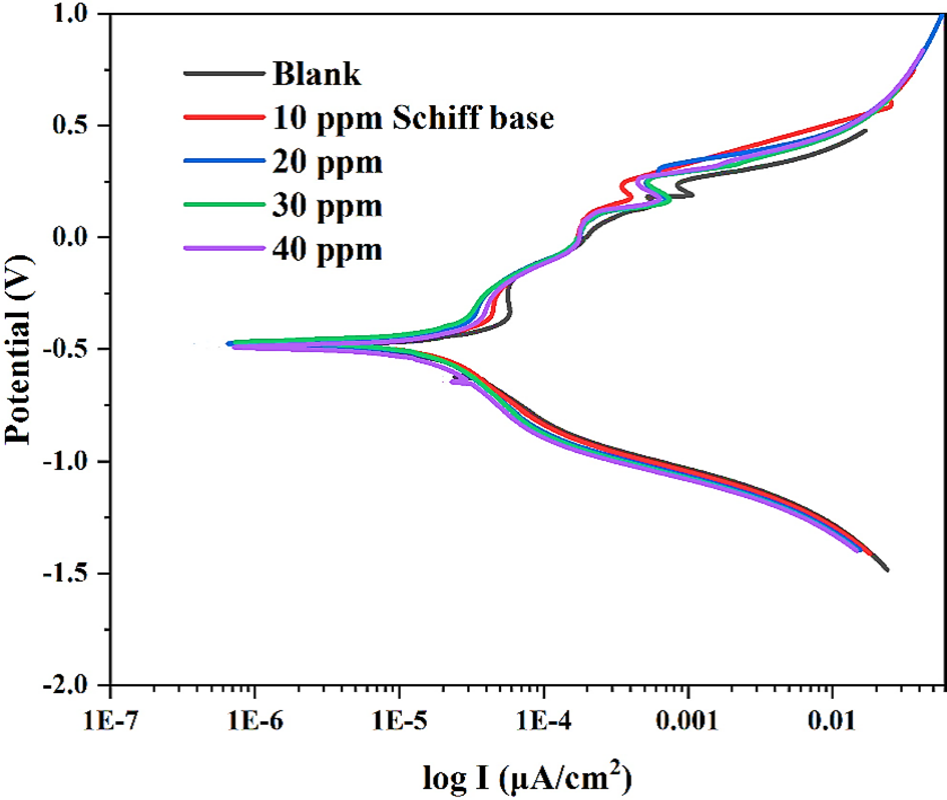
Polarization curve of the Schiff base.

**Figure 18:**
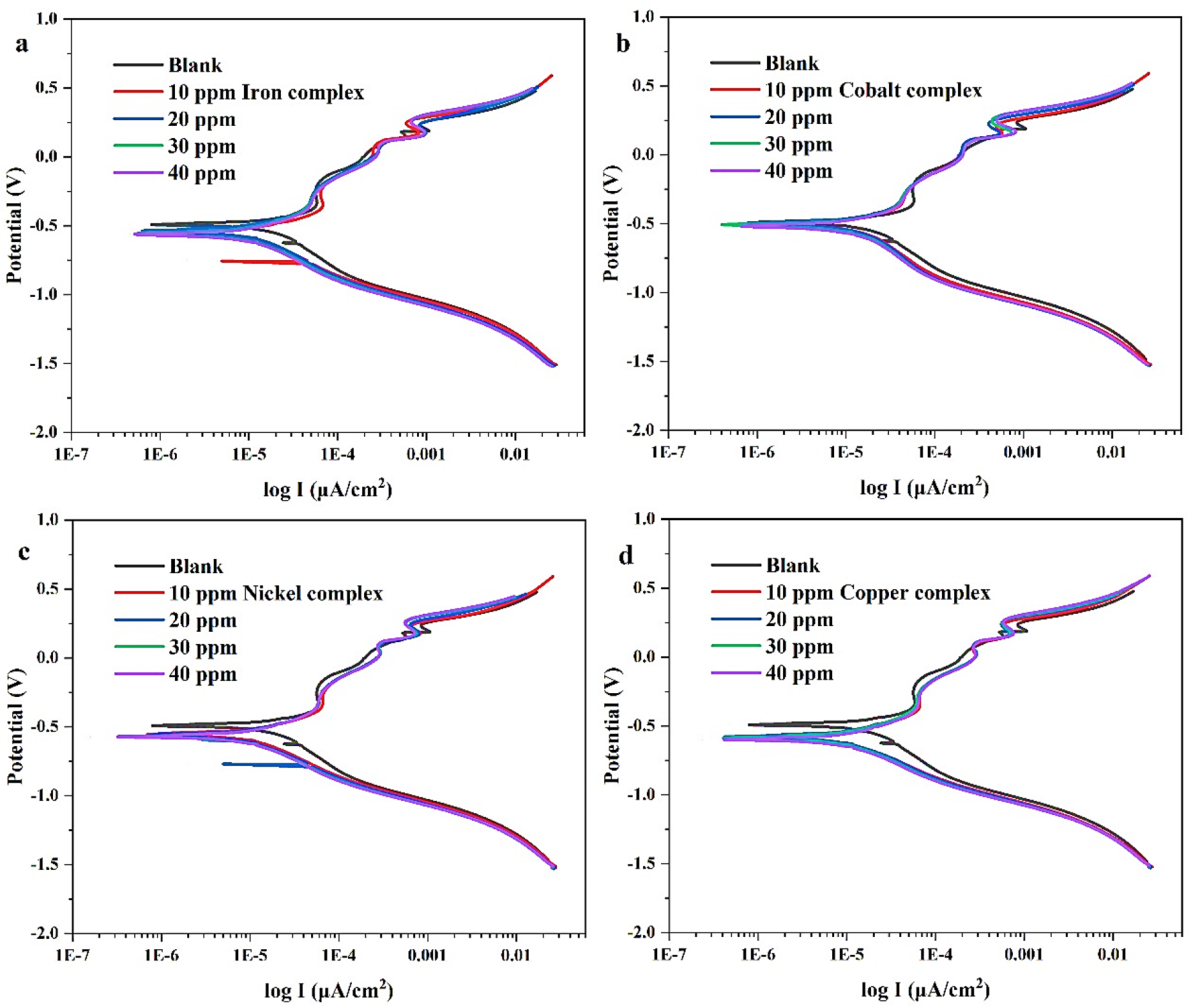
Polarization curve of the metal complexes: (**a**) Fe-complex, (**b**) Co-complex, (**c**) Ni-complex and (**d**) Cu-complex.

Potentiodynamic measurements were executed for a mild steel sample in a 3% aqueous solution of NaCl. The obtained polarization curve was used to detect the corrosion current, and then the corrosion rate of the sample was calculated. Initially, the corrosion current and corrosion rate of steel were recorded in a 3% NaCl solution without an inhibitor as a blank solution. Meanwhile, the effect of compounds on the corrosion rate of steel was determined by applying a potentodynamic scan and observing the corrosion current. Moreover, the experiment was conducted with various concentrations of the Schiff base and its metal complexes (10–40 ppm) to measure the impact of increasing concentration on corrosion current. Figures 17 and 18 display the corrosion potential (E_corr_) and corrosion current (I_corr_), while corrosion rate (CR), inhibition efficiency (IE), and all data are shown in Table 7.

**Table 7:** Schiff base and metal complexes corrosion parameters.

	Concentration (ppm)	I_corr_ (μA cm^−2^)	E_corr_ (mV)	CR (mpy)	IE (%)
Blank		35.42	− 617	16.36	
Schiff base	10	34.55	− 476	15.96	15.38
Iron complex		31.67	− 554	14.63	15.47
Cobalt complex		34.38	− 504	15.89	15.39
Nickel complex		31.04	− 554	14.34	15.48
Copper complex		30.74	− 587	14.20	15.49
Schiff base	20	33.5	− 473	15.47	15.41
Iron complex		29.31	− 536	13.54	15.53
Cobalt complex		33.17	− 499	15.32	15.42
Nickel complex		27.44	− 600	12.68	15.59
Copper complex		26.93	− 582	12.44	15.60
Schiff base	30	32.41	− 467	14.97	15.44
Iron complex		28.02	− 558	12.94	15.57
Cobalt complex		31.75	− 512	14.66	15.46
Nickel complex		26.26	− 573	12.13	15.62
Copper complex		25.91	− 588	11.97	15.63
Schiff base	40	30.83	− 493	14.24	15.49
Iron complex		26.67	− 566	12.32	15.61
Cobalt complex		29.70	− 519	13.72	15.52
Nickel complex		25.24	− 573	11.66	15.65
Copper complex		25.13	− 599	11.61	15.65

The corrosion current of the steel sample in the absence of an inhibitor appears at 35.42 μA cm^−2^, with corrosion potential of − 617 mV, and then the corrosion rate of 16.36 mpy is observed in Table 7. Meanwhile, the effect of adding Schiff base on steel's corrosion current is shown in Fig. 17. It is noted that in the presence of Schiff base, the curve of polarization is shifted to the left-hand side, which elucidates that the corrosion current records a smaller value after the addition of Schiff base. Moreover, the polarization curve after adding 10 ppm Schiff base shows 34.55 μA cm^−2^ corrosion current and – 476 mV corrosion potential, while the corresponding corrosion rate is observed to be 15.96 mpy (Fig. 17). Meanwhile, the addition of 40 ppm of Schiff base reduces the corrosion rate value to 14.24 mpy, and its polarization curve displays the current density and corrosion potential at 30.83 μA cm^−2^ and − 493 mV, respectively (Table 7).

In respect of metal complexes, the polarization curves obtained from the addition of the compounds at concentrations of 10, 20, 30 and 40 ppm are displayed in Fig. 18. It is clear that the complexes minimize the corrosion current more than the Schiff base. Besides, a decline in corrosion current is accompanied by an increase in the efficiency of corrosion inhibition. In this regard, Table 7 exhibits the corrosion rates at 40 ppm for cobalt complex, iron complex, nickel complex, and copper complex, which are found to be 13.32, 12.32, 11.66 and 11.61 mpy, respectively.

**Figure 19:**
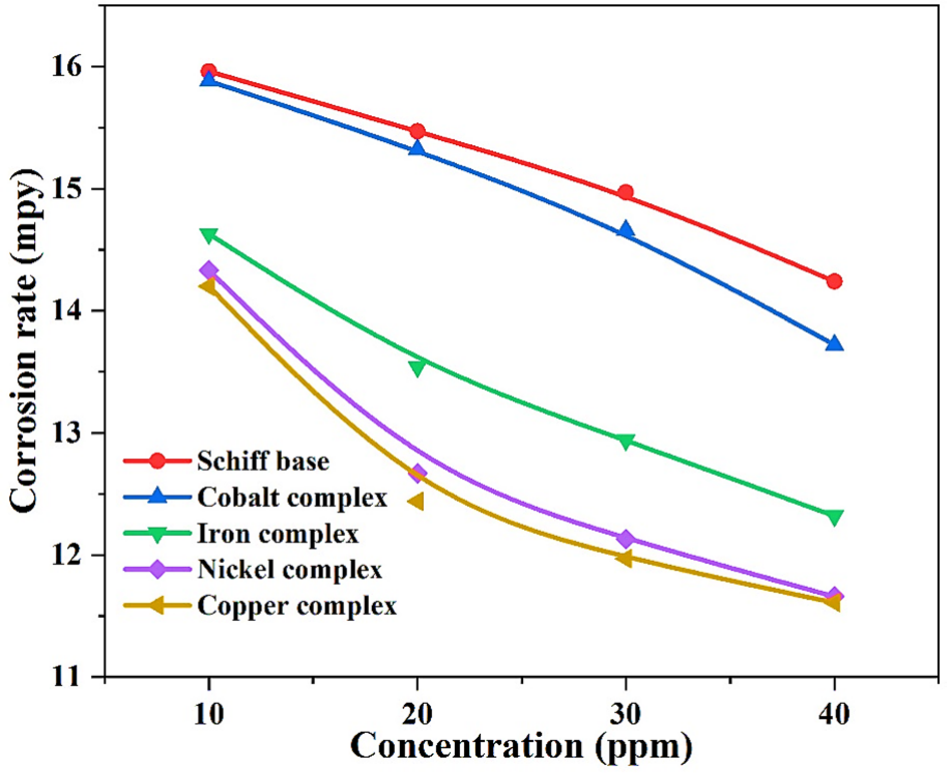
The effect of compounds on corrosion rate with different concentrations.

**Figure 20:**
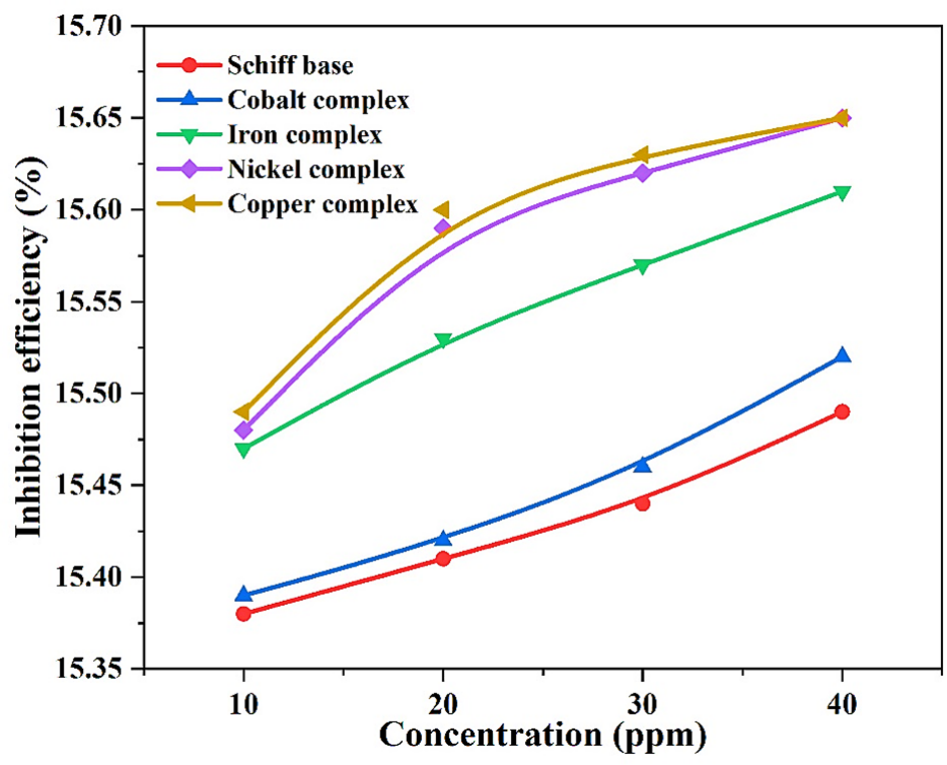
The compounds inhibition efficiencies at different concentrations.

The comparison between the effect of the Schiff base and its derivatives on the corrosion rate (Fig. 19) shows that the corrosion rate of steel is higher in the case of the Schiff base when compared to its complexes. In addition, the complexes increase the corrosion inhibition value more than the Schiff base, and Fig. 20 displays that the copper complex has the greatest corrosion inhibition.

Steel’s corrosion rate order in the presence of inhibitor

$${\mathbf{Schiff}} \, {\mathbf{base}} > {\mathbf{Co}} {-} {\mathbf{complex}} > {\mathbf{Fe}} {-} {\mathbf{complex}} > {\mathbf{Ni}} {-} {\mathbf{complex}} > {\mathbf{Cu}} {-} {\mathbf{complex}}$$

Inhibitor efficiency order

$${\mathbf{Cu}} {-} {\mathbf{complex}} \approx {\mathbf{Ni}} {-} {\mathbf{complex}} > {\mathbf{Fe}} {-} {\mathbf{complex}} > {\mathbf{Co}} {-} {\mathbf{complex}} > {\mathbf{Schiff}} \, {\mathbf{base}}$$

**Figure 21:**
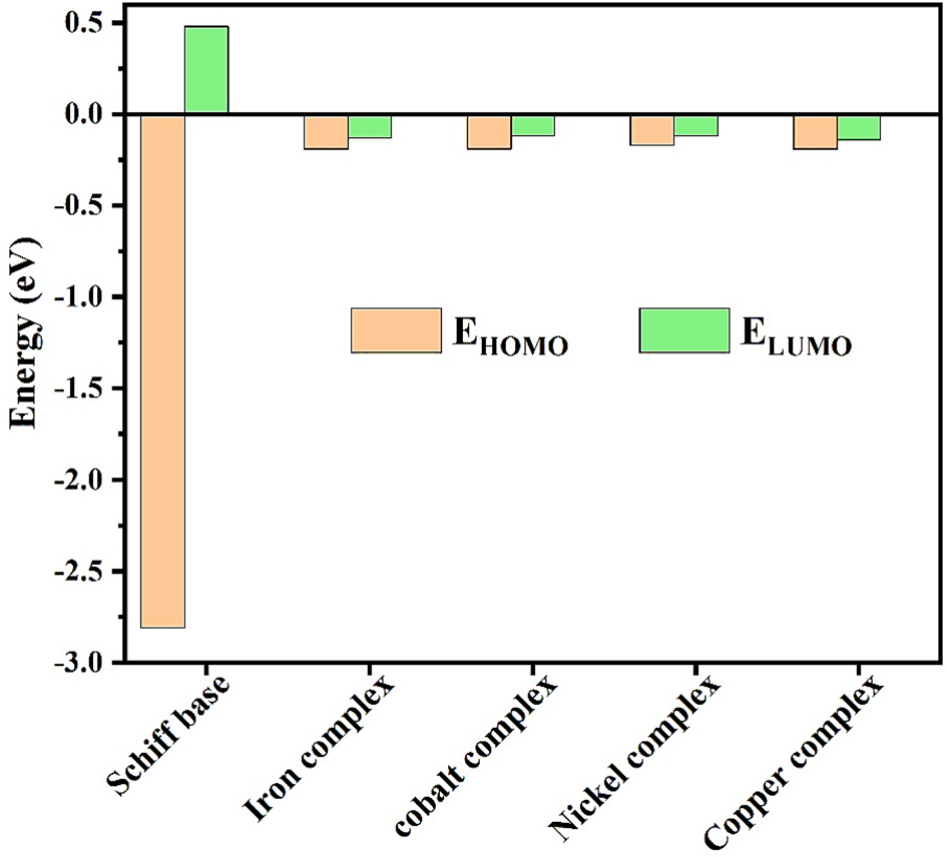
HOMO, LUMO values of Schiff base and its metal complexes.

The previous results go well with the acquired data from molecular modeling calculations, as the ability of organic compounds to donate electrons to metal orbitals and receive electrons from metal surfaces measures the efficiency of compounds as organic inhibitors^54,55^. Meanwhile, Fig. 21 shows that the metal complexes show a rise in ${\text{E}}_{\text{HOMO}}$ from the observed value of the Schiff base, while the Schiff base has a LUMO orbital at a higher energy level than its complexes.

**Figure 22:**
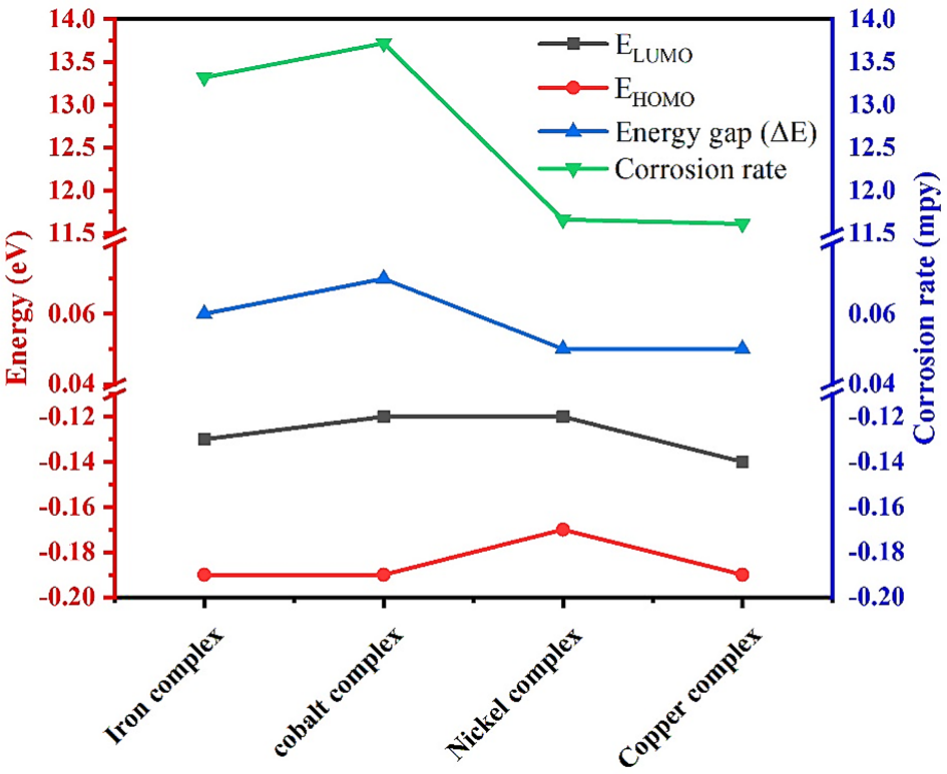
The relation between corrosion rate and energy gap of complexes.

Therefore, the Schiff base has less potential to bind to the steel surface, whether by offering or gaining electrons, due to its HOMO and LUMO energy level. Moreover, the greater the decline in the energy gap value, the better the recorded inhibition efficiency. Meanwhile, Fig. 22 displays that the Cu and Ni complex has the lowest energy gap, thus greatly reducing steel's corrosion rate compared with the Schiff base.

**Figure 23:**
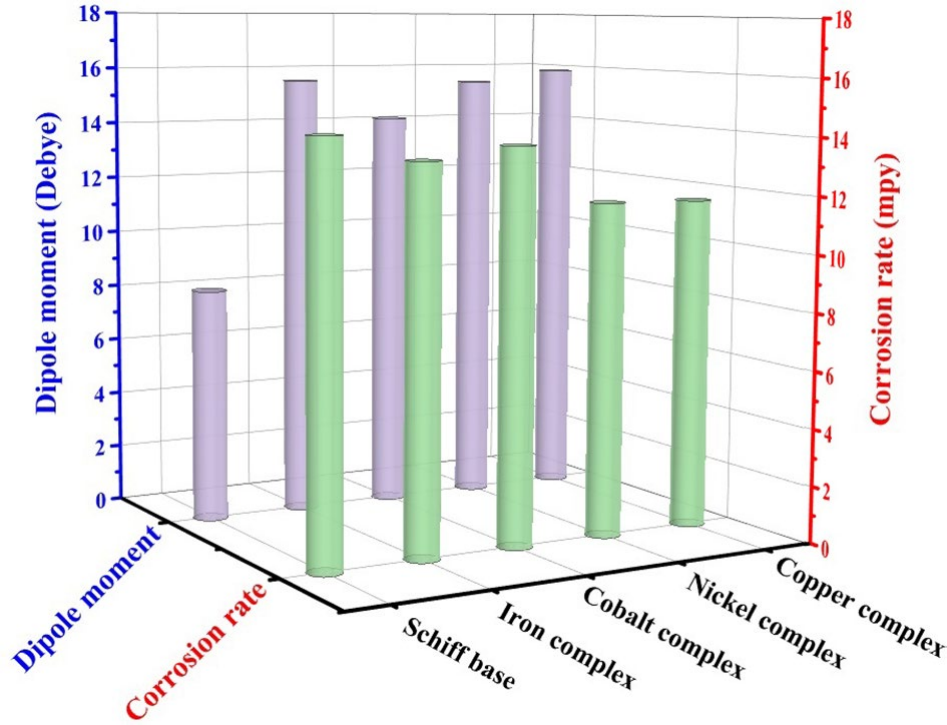
The effect of Schiff base and its complexes dipole moments on corrosion rate.

Regarding the dipole moment, it is applied to estimate the course of action of corrosion inhibition performance. The accumulation of electrons in a molecule is related to the dipole moment, which is an evaluation of charge density in a bond. As a predictor of the manner of corrosion inhibition, it is widely believed that the adsorption process on metal surfaces by compounds with a high dipole moment can cause a greater inhibition impact. Consequently, **Cu complex** can adhere well to steel surfaces if compared to other compounds due to its high dipole moment, and the lowest steel's corrosion rate is observed after its addition (Fig. 23).

## Conclusion

New inorganic inhibitors derived from cefotaxime Schiff base were synthesized and studied using various tools of analysis. The octahedral structure of the achieved complexes from Fe^III^, Co^II^, Ni^II^, and Cu^II^ was proven from spectroscopic and magnetic data. In addition, the DFT calculations were done for the Schiff base and its complexes to evaluate their quantum mechanics parameters. These parameters indicate that the Schiff base has a higher energy gap and hardness than metal complexes. Meanwhile, the observed results from potentiodynamic polarization scans of a steel sample display that the corrosion current is declining after the addition of inhibitors in a 3% NaCl solution. Moreover, the prepared inorganic inhibitors exhibit higher corrosion resistance when compared to the Schiff base.

## Data Availability

All data generated or analysed during this study are included in this published article.

## References

[1] Pradityana A (2016). Inhibition of corrosion of carbon steel in 35% NaCl solution by myrmecodia pendans extract. Int. J. Corros..

[2] Revie RW (2008). Corrosion and Corrosion Control: An Introduction to Corrosion Science and Engineering.

[3] Roberge PR (2019). Handbook of Corrosion Engineering.

[4] Lukovits I, Kalman E, Zucchi FJC (2001). Corrosion inhibitors—correlation between electronic structure and efficiency. Corrosion.

[5] Hooshmand Zaferani S (2013). Application of eco-friendly products as corrosion inhibitors for metals in acid pickling processes—a review. J. Environ. Chem. Eng..

[6] Jones DAJC (1996). Principles and prevention. Corrosion.

[7] Zhao J, Chen G (2012). The synergistic inhibition effect of oleic-based imidazoline and sodium benzoate on mild steel corrosion in a CO2-saturated brine solution. Electrochim. Acta.

[8] Dutta A (2015). Correlating electronic structure with corrosion inhibition potentiality of some bis-benzimidazole derivatives for mild steel in hydrochloric acid: Combined experimental and theoretical studies. Corros. Sci..

[9] Zhang SG (2005). QSAR study on N-containing corrosion inhibitors: Quantum chemical approach assisted by topological index. J. Mol. Struct. (Thoechem.).

[10] Simescu-Lazar F (2023). Thymus satureoides oil as green corrosion inhibitor for 316L stainless steel in 3% NaCl: Experimental and theoretical studies. Lubricants.

[11] Arslan T (2009). Quantum chemical studies on the corrosion inhibition of some sulphonamides on mild steel in acidic medium. Corros. Sci..

[12] Ebenso EE (2021). Molecular modelling of compounds used for corrosion inhibition studies: A review. Phys. Chem. Chem. Phys..

[13] Aljourani J, Raeissi K, Golozar MA (2009). Benzimidazole and its derivatives as corrosion inhibitors for mild steel in 1M HCl solution. Corros. Sci..

[14] El-Haddad MN (2013). Chitosan as a green inhibitor for copper corrosion in acidic medium. Int. J. Biol. Macromol..

[15] Ou H-H, Tran QTP, Lin P-H (2018). A synergistic effect between gluconate and molybdate on corrosion inhibition of recirculating cooling water systems. Corros. Sci..

[16] Bentiss F (2009). Corrosion control of mild steel using 3,5-bis(4-methoxyphenyl)-4-amino-1,2,4-triazole in normal hydrochloric acid medium. Corros. Sci..

[17] Obot IB (2019). Theoretical and experimental investigation of two alkyl carboxylates as corrosion inhibitors for steel in acidic medium. J. Mol. Liquids.

[18] Noor EA, Al-Moubaraki AH (2008). Thermodynamic study of metal corrosion and inhibitor adsorption processes in mild steel/1-methyl-4[4′(-X)-styryl pyridinium iodides/hydrochloric acid systems. Mater. Chem. Phys..

[19] Pradityana A, Sulistijono S, Shahab AJAMR (2015). The influence of adding bio inhibitor sarang semut (Myrmecodia Pendans) to carbon steel API 5L Grade B in solution of HCl 1 M. Adv. Mater. Res..

[20] Al-Zoubi W (2020). Recent advances in hybrid organic-inorganic materials with spatial architecture for state-of-the-art applications. Progress Mater. Sci..

[21] Ramezanzadeh M, Bahlakeh G, Ramezanzadeh B (2019). Elucidating detailed experimental and fundamental understandings concerning the green organic-inorganic corrosion inhibiting molecules onto steel in chloride solution. J. Mol. Liquids.

[22] Sanaei Z, Shahrabi T, Ramezanzadeh B (2017). Synthesis and characterization of an effective green corrosion inhibitive hybrid pigment based on zinc acetate-*Cichorium intybus* L. leaves extract (ZnA-CIL.L): Electrochemical investigations on the synergistic corrosion inhibition of mild steel in aqueous chloride solutions. Dyes Pigments.

[23] Salehi E, Naderi R, Ramezanzadeh B (2017). Synthesis and characterization of an effective organic/inorganic hybrid green corrosion inhibitive complex based on zinc acetate/Urtica Dioica. Appl. Surface Sci..

[24] Anacona JR, Calvo J, Almanza OA (2013). Synthesis, spectroscopic, and magnetic studies of mono- and polynuclear Schiff base metal complexes containing salicylidene-cefotaxime ligand. Int. J. Inorg. Chem..

[25] Reiss A (2014). Transition metal(II) complexes with cefotaxime-derived schiff base: Synthesis, characterization, and antimicrobial studies. Bioinorg. Chem. Appl..

[26] Anacona JR (2021). Ceftriaxone-based Schiff base transition metal(II) complexes. Synthesis, characterization, bacterial toxicity, and DFT calculations. Enhanced antibacterial activity of a novel Zn(II) complex against *S. aureus* and *E. coli*. J. Inorg. Biochem..

[27] Al-Masoudi W, Saeed S (2020). Synthesis and antibacterial activity of new cefotaxime derivatives. Int. J. Pharmaceut. Res..

[28] Reiss A (2017). New metal(II) complexes with ceftazidime Schiff base. J. Therm. Anal.Calorim..

[29] Fu Y (2022). Method improving for isolation and characterization of allergy-inducing polymer impurities in cefotaxime sodium medicines. Pharmaceut. Ind..

[30] Yousif EI (2012). New monomeric (CoII, NiII, CuII and ZnII) metal complexes of a bidentate schiff-base ligand; synthesis, characterisation and biological studies. J. Al-Nahrain Univ. Sci..

[31] Abdulghani AJ, Hussain RK (2015). Synthesis and characterization of schiff base metal complexes derived from cefotaxime with 1H-indole-2,3-dione (Isatin) and 4-N, N-dimethyl-aminobenzaldehyde. Open J. Inorg. Chem..

[32] Anacona JR, Osorio I (2008). Synthesis and antibacterial activity of copper(II) complexes with sulphathiazole and cephalosporin ligands. Trans Metal Chem..

[33] Ejaz S (2019). Designing, synthesis and characterization of 2-aminothiazole-4-carboxylate Schiff bases; antimicrobial evaluation against multidrug resistant strains and molecular docking. BMC Chem..

[34] Patel AK (2021). Mononuclear copper(II) complexes with (Z)-N′-{(2-hydroxynapthalen-1-yl}methylene)acetohydrazide: X-ray single-crystal structures, Hirshfeld analysis, X-band epr spectra, DFT calculations and SOD mimetic activity. Inorg. Chim. Acta.

[35] Mavri J, Grdadolnik J (2001). Proton transfer dynamics in acetylacetone: A mixed quantum-classical simulation of vibrational spectra. J. Phys. Chem. A.

[36] Kazuo N (2009). Infrared and Raman Spectra of Inorganic and Coordination Compounds_ Part A_ Theory and Applications in Inorganic Chemistry.

[37] El-Aarag B (2021). New metal complexes derived from diacetylmonoxime-n(4)antipyrinylthiosemicarbazone: Synthesis, characterization and evaluation of antitumor activity against Ehrlich solid tumors induced in mice. Arab. J. Chem..

[38] Li L (2012). Atomic and Molecular Low-n Rydberg States in Near Critical Point Fluids Advanced Aspects of Spectroscopy.

[39] Kumar U, Chandra S (2010). Biological active cobalt(II) and Nickel(II) complexes of 12-membered hexaaza [N_6_] macrocyclic ligand synthetic and spectroscopic aspects. E-J. Chem..

[40] Chandra S (2015). Coordination mode of pentadentate ligand derivative of 5-amino-1,3,4-thiadiazole-2-thiol with nickel(II) and copper(II) metal ions: Synthesis, spectroscopic characterization, molecular modeling and fungicidal study. Spectrochim. Acta Part A: Mol. Biomol. Spectrosc..

[41] Rao TS, Reddy KL, Lingaiah P (1988). Synthesis and structural studies of complexes of Co(II), Ni(II), Cu(II), Zn(II) and Cd(II) with substituted chalcones. Proc. Indian Acad. Sci. Chem. Sci..

[42] Dianu ML (2010). Transition metal M(II) complexes with isonicotinoylhydrazone-9-anthraldehyde. J. Serbian Chem. Soc..

[43] Shakir M (2011). Synthesis, spectroscopic studies and crystal structure of the Schiff base ligand L derived from condensation of 2-thiophenecarboxaldehyde and 3,3'-diaminobenzidine and its complexes with Co(II), Ni(II), Cu(II), Cd(II) and Hg(II): Comparative DNA binding studies of L and its Co(II), Ni(II) and Cu(II) complexes. Spectrochim. Acta Part A: Mol. Biomol. Spectrosc..

[44] Abd-El-Zahir M (2023). Nanocomposite for enhancement the biological activity of Cu (II)-complex from new cefotaxime derivative. Front. Sci. Res. Technol..

[45] Vidhya V, Austine A, Arivazhagan M (2019). Quantum chemical determination of molecular geometries and spectral investigation of 4-ethoxy-2, 3-difluoro benzamide. Heliyon.

[46] Jone-Kirubavathy S, Chitra S (2017). Structural, theoretical investigations and biological evaluation of Cu(II), Ni(II) and Co(II) complexes of mercapto-pyrimidine schiff bases. J. Mol. Struct..

[47] Sebastian SHR (2016). Spectroscopic, quantum chemical studies, Fukui functions, in vitro antiviral activity and molecular docking of 5-chloro-N-(3-nitrophenyl)pyrazine-2-carboxamide. J. Mol. Struct..

[48] Nataraj A, Balachandran V, Karthick T (2013). Molecular orbital studies (hardness, chemical potential, electrophilicity, and first electron excitation), vibrational investigation and theoretical NBO analysis of 2-hydroxy-5-bromobenzaldehyde by density functional method. J. Mol. Struct..

[49] Refat MS (2017). Synthesis of new drug model has an effective antimicrobial and antitumors by combination of cephalosporin antibiotic drug with silver(I) ion in nano scale range: Chemical, physical and biological studies. J. Mol. Liquids.

[50] Parr RG (1980). Density Functional Theory of Atoms and Molecules, Horizons of Quantum Chemistry.

[51] Merdas S, Marshes Research Center, I (2021). Synthesis, characterization, biological activity and quantum chemical calculations of new oxadiazole derivatives. Egypt. J. Chem..

[52] Kosar B, Albayrak C (2011). Spectroscopic investigations and quantum chemical computational study of (E)-4-methoxy-2-[(p-tolylimino)methyl]phenol. Spectrochim. Acta A Mol. Biomol. Spectrosc..

[53] Islam N, Ghosh DC (2012). On the electrophilic character of molecules through its relation with electronegativity and chemical hardness. Int. J. Mol. Sci..

[54] Rodriguez JA (2020). Mathematical models generated for the prediction of corrosion inhibition using different theoretical chemistry simulations. Mater. (Basel).

[55] Al-Amiery AA (2014). New coumarin derivative as an eco-friendly inhibitor of corrosion of mild steel in Acid medium. Molecules.

